# Textured NiSe_2_ Film: Bifunctional Electrocatalyst for Full Water Splitting at Remarkably Low Overpotential with High Energy Efficiency

**DOI:** 10.1038/s41598-017-02285-z

**Published:** 2017-05-25

**Authors:** Abdurazag T. Swesi, Jahangir Masud, Wipula P. R. Liyanage, Siddesh Umapathi, Eric Bohannan, Julia Medvedeva, Manashi Nath

**Affiliations:** 1 Department of Chemistry, Missouri University of Science and Technology, Rolla, MO 65409 USA; 2 Materials Research Center, Missouri University of Science and Technology, Rolla, MO 65409 USA; 3 Department of Physics, Missouri University of Science and Technology, Rolla, MO 65409 USA

## Abstract

Herein we have shown that electrodeposited NiSe_2_ can be used as a bifunctional electrocatalyst under alkaline conditions to split water at very low potential by catalyzing both oxygen evolution and hydrogen evolution reactions at anode and cathode, respectively, achieving a very high electrolysis energy efficiency exceeding 80% at considerably high current densities (100 mA cm^−2^). The OER catalytic activity as well as electrolysis energy efficiency surpasses any previously reported OER electrocatalyst in alkaline medium and energy efficiency of an electrolyzer using state-of-the-art Pt and RuO_2_ as the HER and OER catalyst, respectively. Through detailed electrochemical and structural characterization, we have shown that the enhanced catalytic activity is attributed to directional growth of the electrodeposited film that exposes a Ni-rich lattice plane as the terminating plane, as well as increased covalency of the selenide lattice which decreases the Ni(II) to Ni(III) oxidation potential. Thereby, the high efficiency along with extended stability makes NiSe_2_ as the most efficient water electrolyzer known to-date.

## Introduction

Water splitting reactions producing readily useable clean fuel H_2_, has been the focus of major research activities in alternative energy since they are benign in terms of impact on the environment and human health. The oxygen and hydrogen evolution reactions (OER and HER, respectively), an intricate part in water oxidation/reduction, respectively, also plays a crucial role in other alternative energy devices including fuel cells, metal-oxygen batteries and solar water splitting devices^1–3^. Among these the oxygen evolution reaction (OER) occurring at the anode is a major hurdle since it is a kinetically sluggish process that involves 4 electron transfer associated with the formation of dioxygen molecule from water, and requires a large anodic potential^4^. Therefore, this OER half reaction is regarded as the most limiting step for water splitting, and electrocatalysts/photocatalysts are typically used to reduce the applied potentials for O_2_ evolution. The most commonly used high-efficiency catalysts are the precious metal oxides (IrO_x_, RuO_x_) which exhibit some of the lowest overpotential for practical current densities^5–12^. In addition significant advances have been recently made in identifying OER electrocatalysts^13^ based on transition metal compounds including alloys (often containing significant amounts of Ni, Co or Fe)^5^, oxide/hydroxide^6–8^, phosphide^9^, spinels^5, 10, 11^ and perovskite oxides^5, 12^. Among these, Ni-based oxides, oxyhydroxides, and hydroxides, along with Ni-Fe based oxyhydroxides have shown the most potent catalytic efficiencies with performance closer to that of precious metal oxides^14–17^. Very recently transition metal chalcogenides has been receiving increasing interest as OER and HER catalysts^18–21^. Compared to the oxides, the chalcogenides are expected to have more covalency, smaller bandgaps, and better band alignment with water redox levels for efficient charge transfer, thus promoting better catalytic efficiency. In particular Ni- and Co-based chalcogenides have shown great promise for water electrolysis in alkaline medium^18–25^. Indeed we have identified a nickel selenide, Ni_3_Se_2_ which shows a very low onset potential and overpotential at 10 mA cm^−2^ for oxygen evolution in alkaline medium^20^. Other groups have also reported chalcogenides such as NiS_x_ as a bifunctional HER and OER active catalysts^18^. The bifunctional electrocatalysts that can be applied both at the cathode and anode and produce hydrogen and oxygen, respectively, are highly appealing technologically for water electrolysis^22^.

In this article we report the highly efficient OER and HER catalytic activity of electrodeposited pure NiSe_2_ films and emphasize the importance of film orientation and growth conditions on the catalytic activity. The OER catalytic activity in these films with the onset potential for O_2_ evolution at 1.36 V (*vs* RHE) and overpotential at 10 mA cm^−2^ at 140 mV in alkaline medium, was observed to be superior to any other OER electrocatalysts reported till date including state-of-the-art precious metal oxides, transition metal oxides, and other nickel chalcogenides. There has been another report of the OER catalytic activity of NiSe_2_ (coated with native oxide layer) *albeit* with much higher overpotential at 10 mA cm^−2^ (~290 mV)^18, 23^. This difference can be attributed to several factors including the surface chemistry, phase purity and the preferential growth direction which influences exposure of the catalytic sites to the electrolyte as discussed below. The later proposition has been conclusively proven in this manuscript whereby, we have grown randomly oriented NiSe_2_ grains through hydrothermal methods and have shown their catalytic activity for OER to be less efficient than the electrodeposited oriented films, thereby providing a very valuable insight for the OER electrocatalysts. The onset potential for H_2_ evolution was also very low as compared to other non-Pt based HER electrocatalyst. The NiSe_2_ bifunctional electrocatalyst reported here could effectively split water at 1.43 V and achieve an electrolysis energy efficiency of 83% producing current density as high as 100 mA cm^−2^, which is the highest that has been obtained so far for a bifunctional water electrolyzer. Electrodeposition being a facile, direct method to grow large area binder-free catalytic films, and the experimental proof that films with preferred orientation will show enhanced catalytic activity indeed highlights the novelty of this report.

## Results and Discussion

### Structural and Morphological Characterization

**Figure 1:**
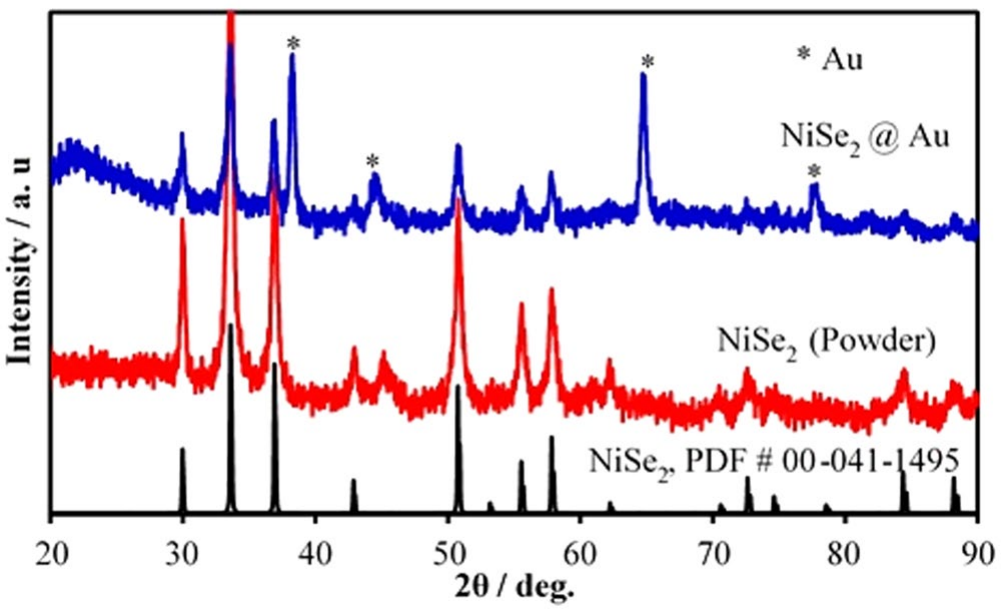
PXRD pattern of electrodeposited NiSe_2_ @ Au (blue) and NiSe_2_ powder (red) showing the presence of NiSe_2_ (PDF # 00-041-1495).

As mentioned above and described in the experimental section in Supporting Information, NiSe_2_ electrocatalysts were grown by two methods – electrodeposition on Au-coated glass and by hydrothermal techniques. The powder X-ray diffraction patterns (PXRD) of as-synthesized NiSe_2_ films electrodeposited on Au-coated glass substrates as well as NiSe_2_ powder synthesized hydrothermally showed peaks that matched well with standard NiSe_2_ (PDF # 00-041-1495) (Fig. 1). The electrodeposited film also showed peaks characteristic of Au arising from the underlying substrate. The peaks were considerably broader indicating that the films were composed of nanostructured grains. Using the Scherrer formula (See Supplementary Information), average particle size in the electrodeposited film was estimated as 30.0 nm.

**Figure 2:**
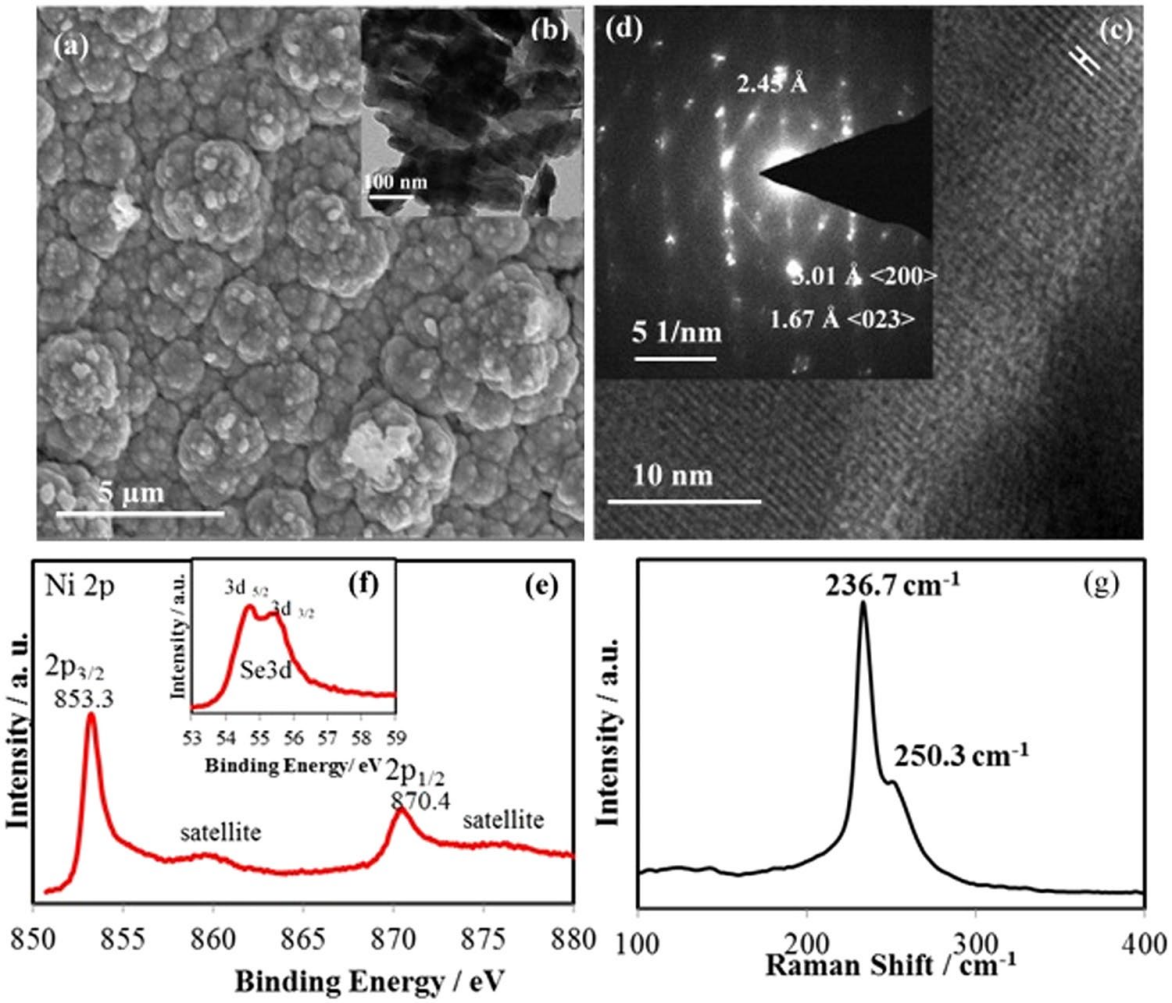
SEM (**a**) and TEM (**b**) images of electrodeposited NiSe_2_, (**c**) HRTEM image of the catalyst showing﻿ lattice spacing of 2.45 A corresponding to <211> lattice planes, (**d**) Typical SAED pattern showing characteristic diffraction spots, (**e**) XPS spectra of the catalyst showing the Ni 2p peaks, (**f**) XPS peaks corresponding to Se 3d. (**g**) Raman spectrum of the catalyst.

For the electrodeposited films, the catalyst loading was measured by weighing the electrode before and after electrodeposition, while the thickness of the catalytic film was estimated from 3-dimensional profiling (Supplementary Fig. 1). Table 1 lists the typical catalyst loading and average thickness of the electrodeposited film. The morphology of the as-synthesized films was investigated through detailed SEM and TEM studies (Fig. 2a and b) which showed that they were indeed composed of nanostructured grains. The TEM and HRTEM images, Fig. 2b and c, respectively, suggests that the film contained mainly nanocrystallites where individual grains were single crystalline showing lattice fringes corresponding to 〈211〉 planes of NiSe_2_. Selected area electron diffraction (SAED) collected from these nanocrystallites showed diffraction spots corresponding to 〈200〉 and 〈023〉 lattice planes of NiSe_2_ (Fig. 2d). The NiSe_2_ granules were free of any amorphous or heterogeneous coating on the surface as apparent from the TEM images, also confirming the purity of these nanograins. The composition of the electrodeposited films were confirmed through EDS (Supplementary Fig. 2) which confirmed the presence of Ni and Se in the film with an approximate ratio of 1:2 and validated the composition as NiSe_2_. The XPS binding energy observed at 853.3 and 870.4 eV as shown in Fig. 2e, corresponds well to the Ni 2p_3/2_ and Ni 2p_1/2_ respectively, similar to that obtained from NiSe_2_^26, 27^, while, binding energies of Se 3d_5/2_ and Se 3d_3/2_ observed at 54.0 eV and 54.6 eV (Fig. 2f), respectively were similar to the reported values for nickel selenides^20, 23^. It should be noted that the peaks corresponding to metal oxide and/or selenium oxide were not observed in the XPS spectrum, indicating that the electrodeposited films were devoid of oxidic impurities.

**Table 1:** Electrochemical parameters of the NiSe_2_ catalysts measured in 1.0 M KOH.

Catalyst	Loading/mg/cm^2^	ECSA/cm^2^	RF	Onset potential/V^a^	η to 10 mAcm^−2^/V^a^	Tafel slope/mV dec^−1^
NiSe_2_/Au	0.13	4.7	16.6	1.36	0.14	48.7
NiSe_2_ (powder)/CFP	1.40	—	—	1.38	0.22	56.6
RuO_2_/Au	—	—	—	1.43	0.32	121.1

^a^Potential reported with respect to RHE.

The electrodeposited films were also studied with Raman spectroscopy which showed peaks at 236.7 and 250.3 cm^−1^ characteristic of NiSe_2_ as shown in Fig. 2g ^28, 29^. The spectrum shows no other major Raman lines attributed to the Se phase and/or Ni–O modes^20^. All of these characterization indicates that the electrodeposited film was purely NiSe_2_ with no discernible impurity phases.

### Electrochemical Performance, OER and HER Catalytic Activities

Electrochemical characterization of the electrodeposited NiSe_2_ films was conducted in N_2_-saturated 1 M KOH (pH 13.6) with a scan rate at 10 mV s^−1^ using a 3-electrode setup. The actual catalytically active region of the material was estimated from the electrochemical surface area (ECSA) measurement (see Supplementary Information, Supplementary Fig. 3) and provided in Table 1.

**Figure 3:**
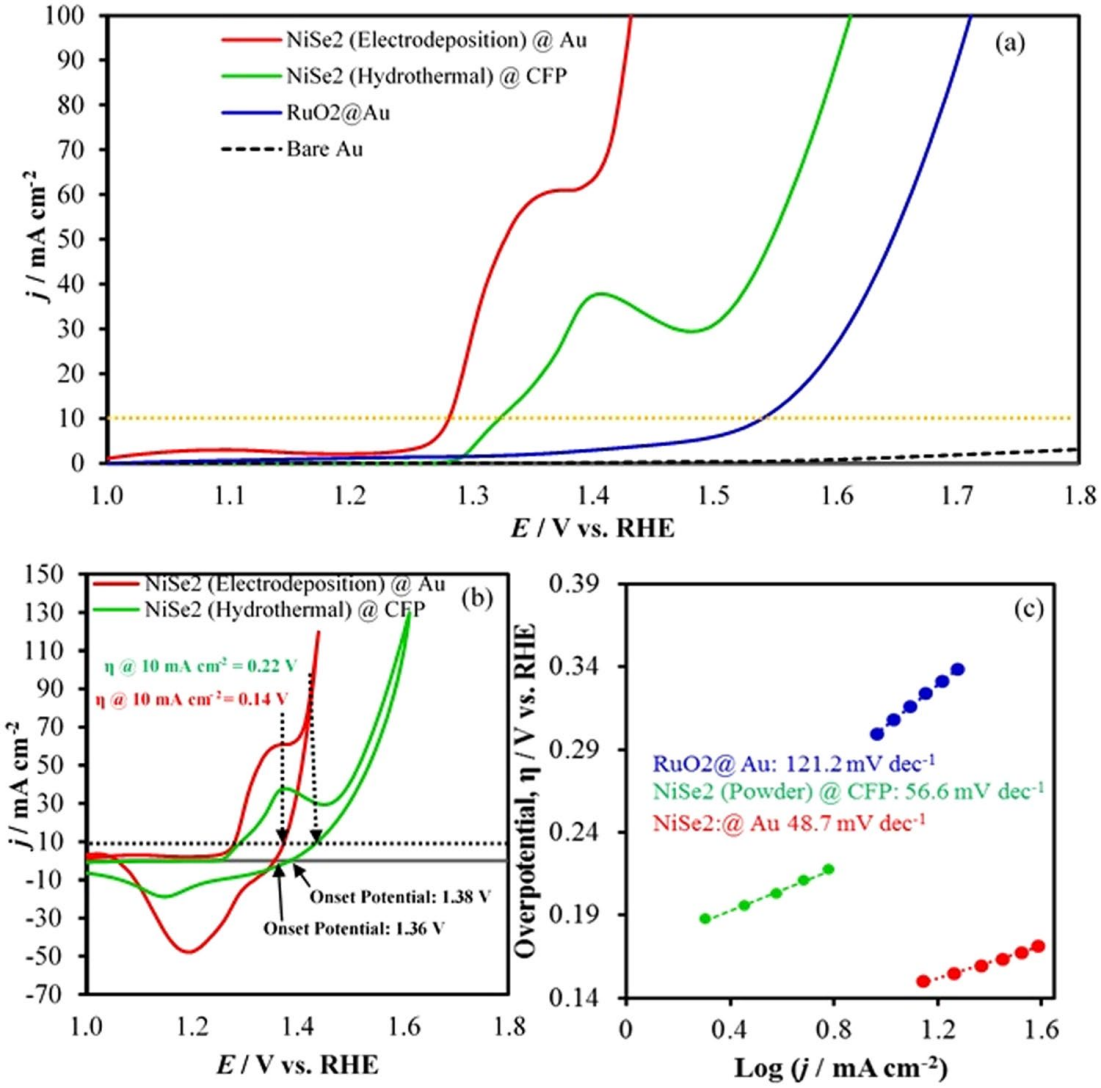
(**a**) LSVs measured at NiSe_2_ (Electrodeposited) @ Au, NiSe_2_ (Hydrothermal) @ CFP, RuO_2_ @ Au and bare Au at in N_2_ saturated 1.0 M KOH solution at a scan rate of 10 mV s^−1^. (**b**) CVs measured under identical condition to estimate the overpotential at 10 mA cm^−2^. (**c**) Tafel plots of catalysts.

The activity of the catalyst for OER was determined from LSVs and CVs (Fig. 3a and b) by measuring the onset potential and overpotential at 10 mA cm^−2^ (*vs* RHE). All the potentials have been reported after iR compensation. Current density with bare Au-glass electrode and RuO_x_ coated electrodes were also measured for comparison. As expected bare Au-coated glass slide showed very minimal catalytic activity for OER, while RuO_2_ coated slide showed an onset potential of 1.43 V (vs. RHE) and overpotential of 320 mV at 10 mA cm^−2^, which matches perfectly with the reported values^8^. It was observed that NiSe_2_ on Au-coated glass electrode exhibits a very low onset potential of about 1.36 V, reaching current density 100 mA cm^−2^ at overpotential of about 200 mV. It must be mentioned here that these value of onset and overpotentials were very reproducible and different batches of NiSe_2_ films on Au-glass showed similar values. The CV for NiSe_2_ showed a large pre-oxidation peak before the increase of current density due to oxygen evolution. For Ni-based OER electrocatalysts, the pre-oxidation peak typically attributed to the Ni^2+^ to Ni^3+^ oxidation has been observed previously. The intensity of the pre-oxidation peak was considerably large, possibly due to exposure of large number of catalytically active sites and high surface roughness that may lead to a high ECSA of the catalyst. Typically, activities of the OER electrocatalysts are benchmarked by comparing the overpotential at current densities of 10 mA cm^−2^ (per geometric surface area), which is considered to be equivalent to 10% solar water-splitting device under 1 sun illumination^30^. In the present case, however, the onset of OER activity as well as the overpotential at 10 mA cm^−2^ was heavily masked by the large pre-oxidation peak and the overpotential at 10 mA cm^−2^ was obtained from the cyclic voltammogram (CV) by analyzing the current density of the reverse scan which corresponds to the OER current only (Fig. 3b). Accordingly, it was observed that a current density of 10 mA cm^−2^ was obtained at an overpotential of 140 mV. This value is much better than the RuO_2_ (320 mV overpotential required to get 10 mA cm^−2^ current density) and is the lowest value reported till date amongst all the known OER electrocatalysts as well as amongst the recently reported chalcogenides (see Supplementary Table 1)^15^. To the best of our knowledge, this is the first time that pure NiSe_2_ has been reported as an active electrocatalyst for OER with such a low onset potential as well as overpotential at 10 mA cm^−2^. To understand the role of catalyst growth conditions, we have also measured the catalytic activity of NiSe_2_ grown through hydrothermal methods. For this purpose, the NiSe_2_ powder was mixed with Nafion and drop-casted on carbon fiber paper (CFP) and used as an anode in the OER set-up (see Supporting Information for details). The CV plot showed that NiSe_2_ powder on CFP was still catalytically active for O_2_ evolution with a low onset potential (1.38 V) and overpotential at 10 mA cm^−2^ (220 mV) (Fig. 3b) indicating that this is indeed an intrinsic property of this material. Noticeably, the catalytic activity of hydrothermally grown pure NiSe_2_ powder was better than previous reports of NiSe_2_^23^, possibly due to the nanostructured morphology of the NiSe_2_ grains as has been frequently obtained with hydrothermal synthesis. Recently we have also shown that nanostructuring increases catalytic activity^31^. However the catalytic activity was inferior to the electrodeposited NiSe_2_ film, thereby, emphasizing the fact that growth conditions indeed play a vital role in determining the catalytic activity of these electrocatalysts (*vide infra*). A small value of Tafel slope of 48.7 mV dec^−1^ for NiSe_2_ compared to RuO_2_ (121.2 mV dec^−1^) confirmed that the catalytic process was very facile. All the parameters defining the electrocatalytic activity of NiSe_2_ has been listed in Table 1.

A common concern with the Ni-based OER electrocatalysts in the alkaline medium is that the catalytic activity is obtained from the oxide-hydroxide phases formed *in situ*. To address this issue, we have intentionally electrodeposited Ni(OH)_2_ films following a reported procedure on Au-coated glass. The electrocatalytic activity of the Ni(OH)_2_ was measured in the same way as described above. It was observed that the onset potential for O_2_ evolution as well as overpotential at 10 mA cm^−2^ was much higher at 1.49 V vs RHE and 330 mV, respectively (Supplementary Fig. 4). This proves that the highly efficient catalytic activity obtained with the electrodeposited NiSe_2_ is definitely an intrinsic property of the selenide itself and not merely arising from the oxide-hydroxide. This claim has been supported by other electrochemical studies (*vide infra*).

The composition of the evolved gas was confirmed to be O_2_ through further electrochemical studies using rotating ring disk electrodes (see Supplementary Information and Supplementary Fig. 5a) and the Faradaic efficiency was observed at about 99.5% at the applied disk potential of 1.4 V (*vs*. RHE) (see Supplementary Information and Supplementary Fig. 5b), that corresponds to about 1.0 mA cm^−2^ disk current density. As the disk voltage increased to 1.46 V (*vs*. RHE), the Faradaic efficiency decayed to 50.2%. This decrease could be attributed to large amounts of oxygen being produced at the disk electrode that cannot be efficiently collected by the Pt ring electrode^20^.

**Figure 4:**
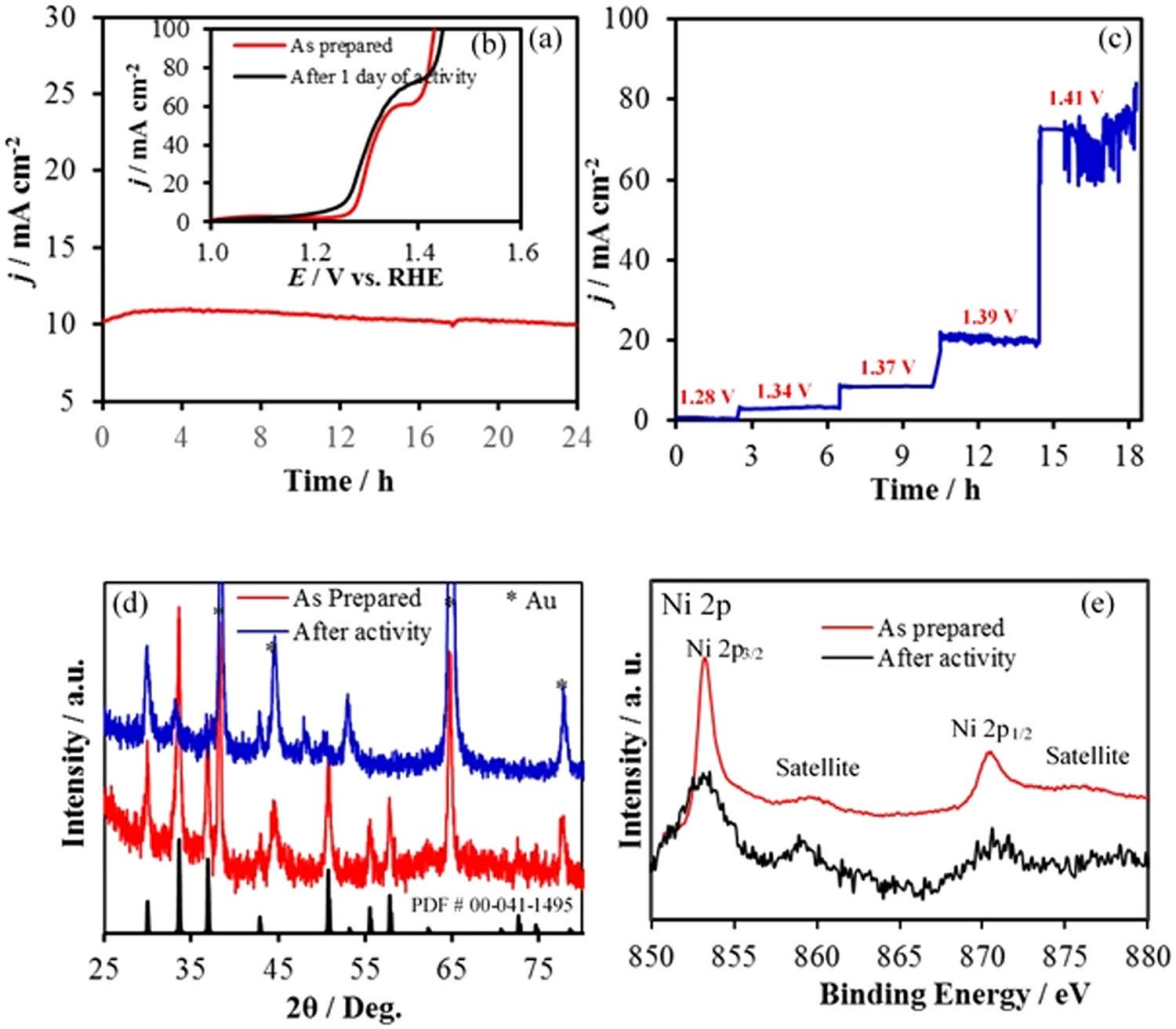
(**a**) Stability study of catalyst under continuous O_2_ evolution at a constant potential to achieve 10 mA cm^−2^ for 24 h, (**b**) LSVs of catalyst in N_2_ saturated 1.0 M KOH before and after chronoamperometry for 24 h, (**c**) Stability of catalyst at different potential by using potential step method. (**d**) XRD patterns before and after current transient experiment for 24 h and (**e**) XPS spectra before and after of stability.

Stability of the catalysts under conditions of continuous O_2_ evolution (in 1.0 M KOH) was investigated using constant potential electrolysis (chronoamperometry) for 24 h where the potential was held at a constant value of (1.37 V *vs* RHE) to deliver 10 mA cm^−2^ per geometric area (Fig. 4a). As can be seen from the chronoamperometry data, there was no degradation of the current density with time indicating extended catalyst stability. It should be noted here that the constant potential of 1.37 V was chosen based on the reverse scan of the CV plot which provided a better estimate of the potential needed for achieving a 10 mA cm^−2^ (Fig. 3b). A separate set of stability studies was conducted by holding the potential constant in several stages of the OER reaction (from onset to rapid O_2_ evolution stage) and the current density was measured at each of these steps for extended period. As can be seen from Fig. 4c, the current density at 1.28 V was almost 0, which can be expected from the LSV curve since it falls below the onset potential. As the potential step was increased to 1.34 and 1.37 V, the current density showed a steady step-wise increase to ~10 mA cm^−2^ where it stayed stable for the duration of the potential step. As the potential step was increased further to 1.39 V and 1.41 V respectively, the current density showed a much steeper increase indicating that OER was indeed the major process in these potential ranges. The current density at 1.41 V was noisy possibly due to evolution of large amounts of O_2_ which led to accumulation of bubbles near the electrode surface and had to be dislodged with mechanical agitation, thereby producing a noisy data. The composition of the film after chronoamperometry and extended periods of continuous O_2_ evolution was analyzed through PXRD (Fig. 4d) and XPS (Fig. 4e) studies which revealed that the films were still predominantly NiSe_2_ even after 24 h of continuous O_2_ evolution. However, a small intensity peak was also observed in the pxrd pattern that corresponds to Ni_0.85_ Se, indicating that there might be some Se loss from the films during extended period of O_2_ evolution leading to formation of a selenium deficient phase. The formation of selenium deficiency on the surface after OER activity was also evidenced further by EDS (Supplementary Fig. 6) as the ratio of Ni to Se increased slightly with progress of reaction.

XPS studies on the other hand, revealed that the Ni and Se (Supplementary Fig. 7) XPS signals were unaltered after the chronoamperometric studies indicating that the catalyst did not undergo a drastic decomposition. Also oxidic peaks were notably absent in the XPS spectra, demonstrating that the catalytic film did not undergo conversion to the oxide, as has been reported for some selenides^18, 23^. The O 1 s signal was also monitored through XPS before and after chronoamperometry (Supplementary Fig. 8) which revealed that there was no evidence of Ni-oxides. In the as-synthesized sample, the O 1 s peak showed only surface adsorbed oxygen. The deconvoluted O 1 s peak after chronoamperometry showed evidence of surface-absorbed oxygen along with physisorbed H_2_O and traces of Se-oxide.

### Mechanism

**Figure 5:**
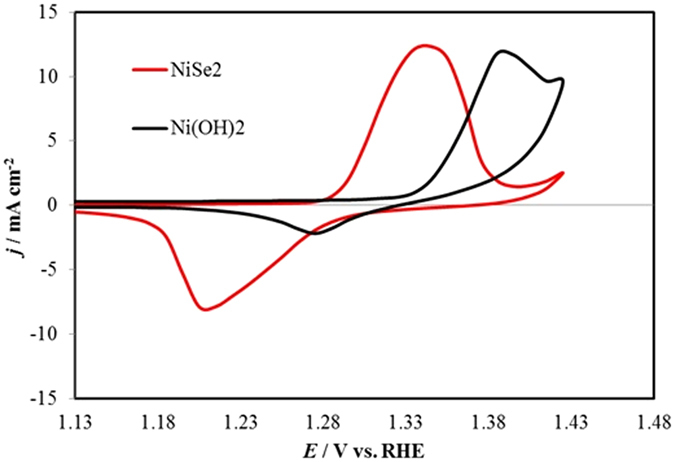
CVs measured at NiSe_2_ and Ni(OH)_2_ catalysts in N_2_ saturated 1.0 M KOH at 10 mV s^−1^. The Ni(OH)﻿_2_ CV has been scaled by a factor of 8.

As was observed with the OER catalytic process, the Ni^2+^ → Ni^3+^ oxidation preceded the oxygen evolution reaction indicating that Ni^3+^ may be the actual catalytic center as has been reported by other researchers for Ni-based OER electrocatalysts^6, 7, 31, 32^. However, in NiSe_2_, the Ni^2+^/Ni^+3^ oxidation potential shows a shift towards lower value as compared to that observed in the Ni-based oxides or oxide-hydroxides as seen from the LSVs. We have probed this point further by collecting cyclic voltammograms (CVs) with electrodes coated with pure NiSe_2_ and Ni(OH)_2_ in 1.0 M KOH with a three electrode set-up. It should be noted here that to check the oxidation peak, the CVs were measured on electrodes prepared with less NiSe_2_ deposition time (5 min), such that the Ni^2+^ → Ni^3+^ oxidation peak was not masked by the stronger OER peak. It was observed that the Ni^2+^ to Ni^3+^ oxidation indeed occurs at much lower potential (1.345 V vs. RHE) compared to that in Ni(OH)_2_ (1.395 V vs. RHE), indicating that the catalytically active species (Ni^3+^) is generated at a lower potential in the NiSe_2_ electrode (Fig. 5). Such kind of downward shift of the oxidation peak potential can be expected by considering the effect of surrounding ligands on the oxidation potential of the central metal atom. In fact, the electronegativity values of Ni (1.91) and Se (2.55) are very close to each other indicating that as the ligands change from oxo-based to seleno-based, the covalent nature of the metal-chalcogen bond increases. The observation of a lower Ni^2+^ to Ni^3+^ oxidation potential in seleno-based coordination environment is also supported by our recent studies with a seleno-based molecular Ni coordination complex containing tetrahedral NiSe_4_ core, similar to that found in most Ni-selenides^33^. Single crystals of this complex showed that Ni^2+^ to Ni^3+^ oxidation occurred at 1.34 V (*vs* Ag|AgCl) in 1.0 M KOH, confirming that the low Ni^2+^ → Ni^3+^ oxidation potential observed in NiSe_2_ is indeed due to the selenide coordination. The increasing covalency in the selenides compared to oxides is also supported from Fajan’s rule^34^. This increase in covalency leads to several important consequences including a decreased bandgap of the selenides as compared to that of oxides (3.5 eV) and an upward shift of the valence band edge. These changes in the orbital energy levels will directly influence their alignment with respect to the water oxidation and reduction levels, which in turn will affect the charge transfer between the catalyst and water. For the water splitting catalysts, one of the most influential factors in light of the electronic band structure is that water oxidation-reduction levels are bracketed within the valence and conduction band edges of the catalyst. In these electrocatalytic systems charge transfer occurs at the semiconductor (catalyst)-electrolyte interface which will be influenced by the relative energy levels of the semiconductor and aqueous electrolyte, and an efficient charge transfer will occur when these two levels are closer in energy, thereby facilitating the catalysis mechanism as proposed above.

**Figure 6:**
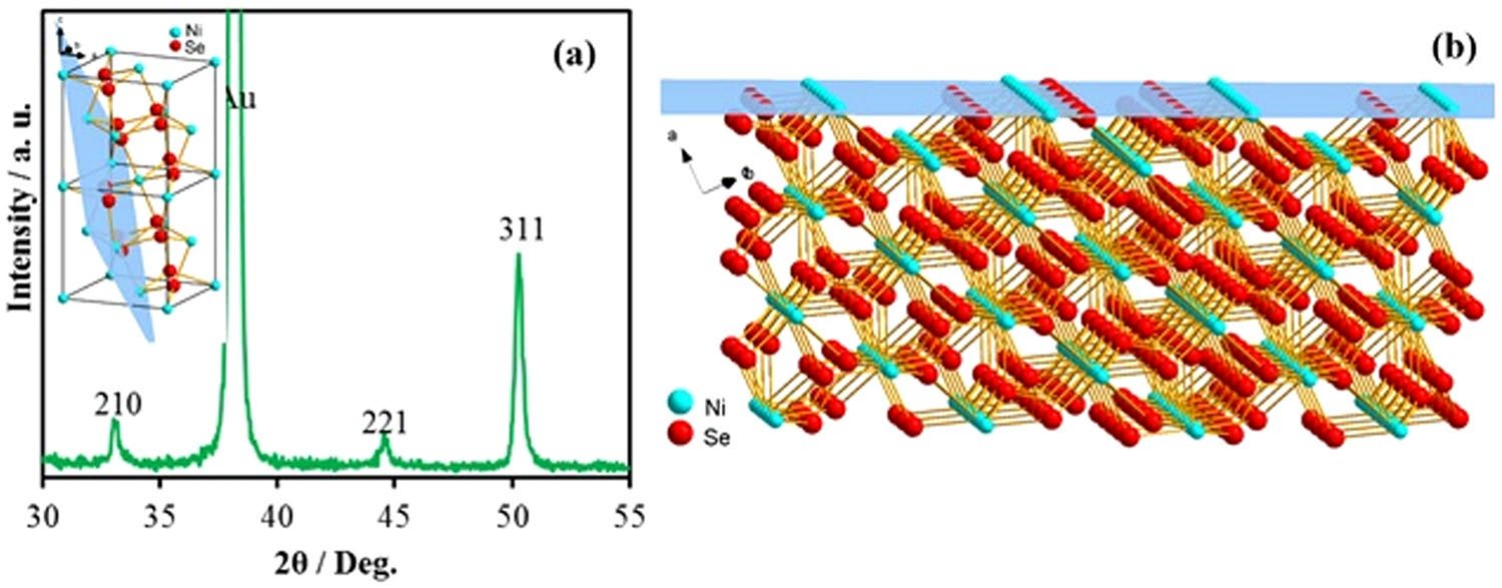
(**a**) Pxrd pattern of the (311) textured NiSe_2_ film. Inset shows the crystal structure with the marked (311) plane. (**b**) An illustration of the NiSe_2_ lattice terminated with the (311) plane showing excess Ni atoms on the surface.

Nickel selenides are an interesting family of compounds and have been researched by solid state chemists for a long time owing to their novel electronic as well as magnetic properties^35^. Several stoichiometries of the nickel selenides has been known including NiSe, NiSe_2_, Ni_3_Se_2_, Ni_3_Se_4_, and Ni_7_Se_8_^36^. Among these, NiSe_2_ has a pyrite structure (space group T_h_^6^(Pa_3_)) with dumbbell-shaped Se_2_ units between two Ni atoms^36^. Most of these nickel selenides are reported to be narrow bandgap semiconductors or semimetals. Previous electronic band structure calculations reported zero bandgap for NiSe_2_ as obtained using GGA, exact exchange hybrid functional, and GW approximation^37^. Another factor that might enhance the catalytic activity is easy access to the active site, which in turn can be influenced by growth direction of the film. Hence, the preferential growth direction of the film was analyzed by studying the texture of the film from PXRD data. Specifically, to perform this kind of texturing study, a symmetric diffraction was utilized, where incident and detector angles were always equal. Under these conditions, diffraction from planes that are oriented parallel to the sample surface was only observed. This is the same general method used for powder samples. However, for a powder, all expected reflections are observed as the powder itself consists of randomly oriented grains. On the other hand, if texturing is present in the sample, it implies that there is a preferential growth direction, and the plane normal to that growth direction will predominate in the diffraction pattern. In this case, the pattern obtained was consistent with (311)-textured NiSe_2_ (Fig. 6) which crystallizes with a primitive cubic structure, penroseite^25^. Substantial (311) texture was evident by the enhanced intensity of the (311) reflection at 2θ~50.5° when compared to the intensity of the (210) reflection at 2θ~33.5° while the underlying Au substrate showed a 〈111〉 orientation as shown in Fig. 6a. In a randomly oriented film, based on the theoretical PXRD generated from the atomic coordinates, one would expect the (311) reflection to be roughly one-third the intensity of the (210) as has been observed in Fig. 1. Our results suggested that (311) planes are preferentially oriented parallel to the growth direction of the film. In other words, the average terminating planes of the film were the 〈311〉 lattice planes of NiSe_2_. Similar texturing studies on the hydrothermally grown NiSe_2_ did not show any preferred orientation. The Au surface with a preferred orientation along the 〈111〉 direction may play a substantial role in determining the growth direction of the film. Similar results has been recently reported by Switzer *et al*. by growing preferentially oriented Cu_2_O grains on Au-coated silicon substrates^38^. Figure 6b shows the (311) plane of the NiSe_2_ lattice and interestingly it can be seen that this crystallographic plane is Ni-rich, and can be considered as predominantly Ni-terminated plane. Since, Ni is the catalytically active center for OER, the anion deficient terminating lattice plane will facilitate the attachment of the hydroxyl anions to the metal center leading to a low onset potential and low Tafel slope. The Ni-rich terminating lattice plane may also reduce Se loss with prolonged OER catalytic activity thereby reinforcing catalyst’s stability. In fact, recently, another group has reported OER electrocatalytic activities of NiSe_2_ in alkaline medium *albeit* with much higher overpotential (250 mV)^23^. They have also shown through *in situ* Raman measurements that the catalytic activity proceeds with the immobilization of OH^−^ on the surface. However, they have also mentioned that their as-prepared catalyst had a surface coating of oxide-hydroxide. That may be the reason the overpotential showed a higher value than the pure NiSe_2_ reported in this study. As we have shown above, the generation of the catalytically active center (Ni^3+^) is much more facile in the chalcogenide lattice than the oxide matrix. The higher conductivity of the inner NiSe_2_ lattice will aid in faster electron transfer leading to higher current densities even at low potentials. Hence, the enhanced intrinsic OER catalytic activity in NiSe_2_ can be explained from the increased covalency in the lattice which affects the electronic band structure of NiSe_2_, band positioning, zero band gap of the material, while the highly efficient catalytic activity of the electrodeposited film can be explained from the preferential growth during electrodeposition. It should be also noted that the catalyst loading in this report was very comparable and in fact lower than that reported for other OER electrocatalysts (Supplementary Table 1). Nevertheless, to rule out any effect of catalyst loading on the OER activity, we have compared the catalytic activity between the electrodeposited 〈311〉 -textured NiSe_2_ film and hydrothermally synthesized NiSe_2_ powder containing identical loading. As can be seen from the CV plot (Supplementary Fig. 9), there was no change of overpotential with variation in catalyst loading, however, the current density increased with increased loading. More importantly, the overpotential for the textured NiSe_2_ film showed the same difference with that obtained for the NiSe_2_ powder, indicating that this difference in activity is intrinsic.

#### Electrocatalytic performance for HER and Overall Water Splitting

**Figure 7:**
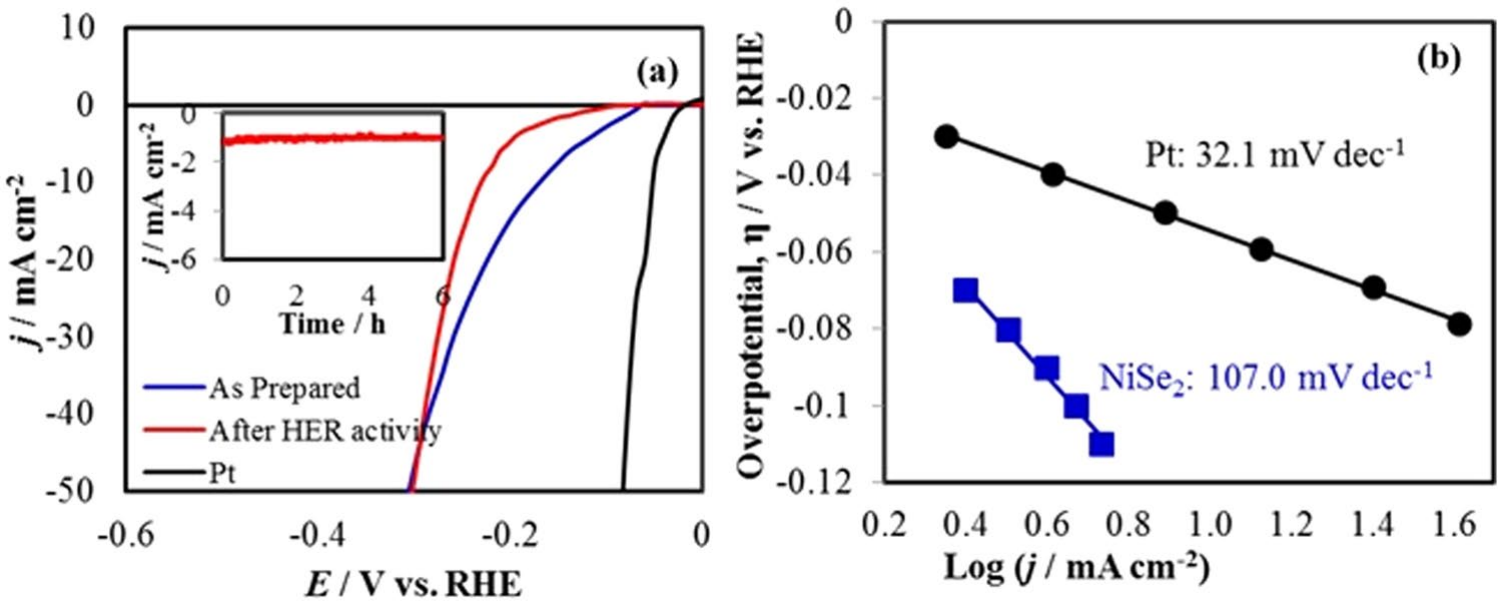
(**a**) LSVs measured at NiSe_2_/Au and Pt in N_2_ saturated 1.0 M KOH solution at a scan rate of 0.01 V s^−1^. Inset shows the HER stability of NiSe_2_. (**b**) Tafel plots of catalysts.

Electrodeposited NiSe_2_ showed considerable catalytic activity for HER in 1 M KOH measured using a three-electrode system with a scan rate of 10 mV s^−1^ and was compared with commercially available Pt electrocatalyst. As shown in the polarization curve (*j*_s_ *vs*. *E*), NiSe_2_ required an overpotential [η_(HER)_] of 170 mV to achieve 10 mA cm^−2^ (Fig. 7a), which is slightly higher than that observed with Pt^39^. A Tafel slope of 107 mV dec^−1^ was obtained for the HER process (Fig. 7b). Non-precious HER catalysts often exhibit Tafel slopes ranging from 40 to 120 mV dec^−1^ ^40–49^.

**Figure 8:**
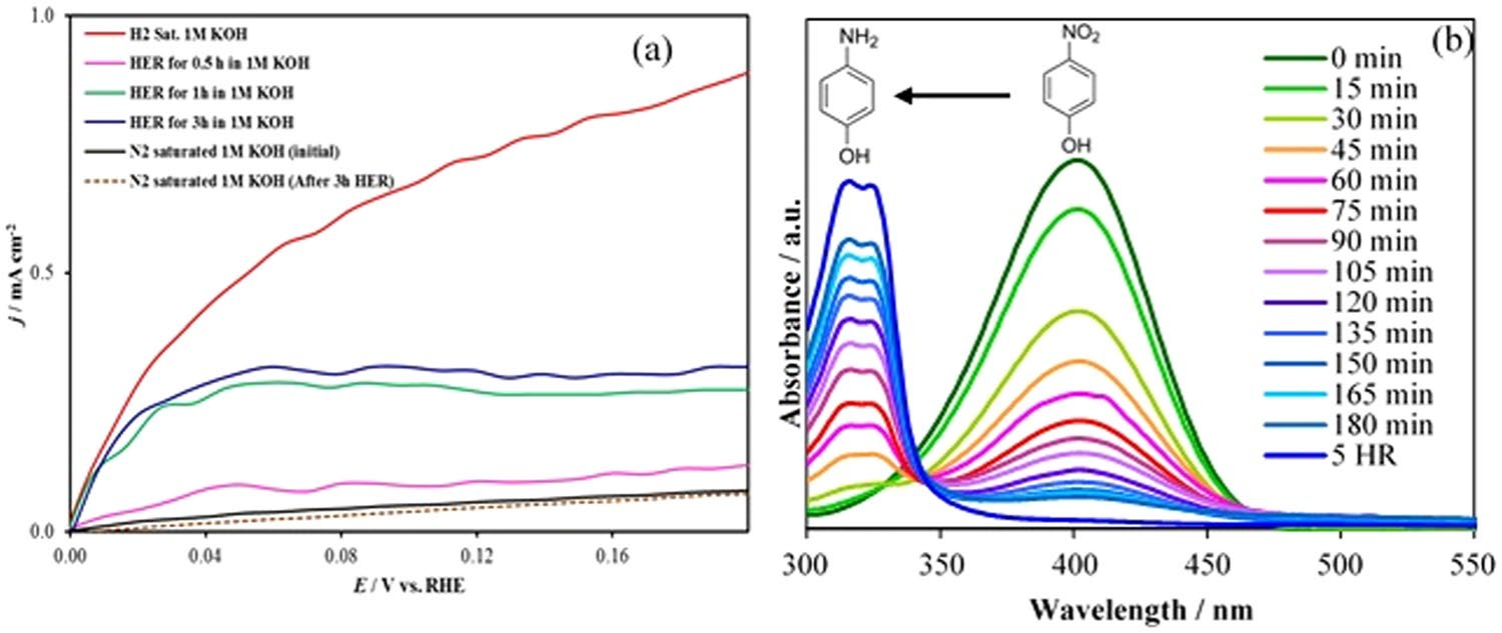
(**a**) Evidence of H_2_ evolution was confirmed by electrochemical oxidation of hydrogen in a Pt electrode. Initially HER was performed at NiSe_2_/Au at a constant potential of −0.2 V *vs* RHE for different periods of time (0.5, 1 and 3 h) under N_2_ saturated and blanketed 1.0 M KOH. The evolved gas was then oxidized with Pt electrode, whereby the oxidation potential and current density confirmed the presence of H_2_. In the absence of H_2_ [before HER, (black solid line) and after purging with N_2_ (reddish dashed line)] the current density was very close to zero. (**b**) Catalytic hydrogenation of p-nitrophenol to p-aminophenol monitored by the change in UV-visible absorbance spectra, confirming the evolution of active hydrogen.

Remarkably, an early onset overpotential (50 mV *vs*. RHE), indicated that the negative current increased rapidly under more cathodic potentials. Such low onset potential places NiSe_2_ amongst the most active non-precious HER catalysts in an alkaline medium^50–52^. Previous report of HER activity with NiSe_2_ corroborates very well with our report signifying the accuracy of the results. Chronoamperometric studies for continuous H_2_ evolution for 6 h showed that the catalyst was also stable for HER activities in 1.0 M KOH for extended time (inset of Fig. 7a). To confirm that the composition of the evolved gas was indeed H_2_ we designed an electrochemical experiment where the evolved gas was oxidized at Pt electrode. Specifically, a series of HER experiments were carried out in 1.0 M KOH, where H_2_ was generated for different time intervals (0.5, 1 and 3 h) at NiSe_2_/Au. After periods of continuous gas evolution for certain periods of time, the evolved gas was oxidized at Pt electrode. As has been shown previously, Pt can electrochemically oxidize H_2_ at 0.0 V^53^. Hence monitoring the H_2_ oxidation potential and oxidation current could confirm the composition of the gas was indeed H_2_. Initially the electrolyte was purged with N_2_ and as expected the current density for H_2_ oxidation at Pt electrode was very close to 0. After the HER reaction was done for 0.5, 1 and 3 h, the oxidation current density increased indicating the increasing concentration of dissolved H_2_ in the electrolyte (Fig. 8). This was further confirmed by measuring the oxidation current after purging the electrolyte with N_2_ after 3 h of HER to get rid of any dissolved gas. As expected, the oxidation current reduced back to the initial value after purging with N_2_. This simple experiment proved conclusively that H_2_ was indeed produced at the NiSe_2_ electrode. The composition of the evolved gas was further confirmed as H_2_ following the well-established catalytic hydrogenation of *para*-nitrophenol (PNP) to *para*-aminophenol (PAP). The hydrogenation reaction was performed as described in supporting information, and the progress of reaction was monitored through UV-Vis spectroscopy since PNP and PAP shows distinctly different λ_max_ values (Supplementary Fig. 10). When the electrolyte was maintained at conditions for HER, it was clearly observed that the absorbance peak due to PNP showed a gradual decrease accompanied by an increase of the PAP absorbance (Fig. 8b) indicating conversion of PNP to PAP. After 5 h, the conversion of PNP to PAP was complete and the solution turned nearly colorless. Two separate control experiments involved passing H_2_ through the reaction mixture in the absence of applied voltage and maintaining a blank electrode (Au-glass) under the HER conditions mentioned above. In both of these control experiments, absorbance of PNP did not show any detectable change even after 5 h. This indicates that not only H_2_ is being produced by NiSe_2_, but it is actually forming catalytically active hydrogen capable of performing liquid phase hydrogenation under ambient conditions. This itself is a significant achievement with long-term technological impact.

The Faradic efficiency for HER process was calculated by quantifying the amount of H_2_ produced and comparing it with the theoretical yield (details has been provided supplementary information). A Faradaic efficiency close to 100% was obtained for HER (Supplementary Fig. 11).

**Figure 9:**
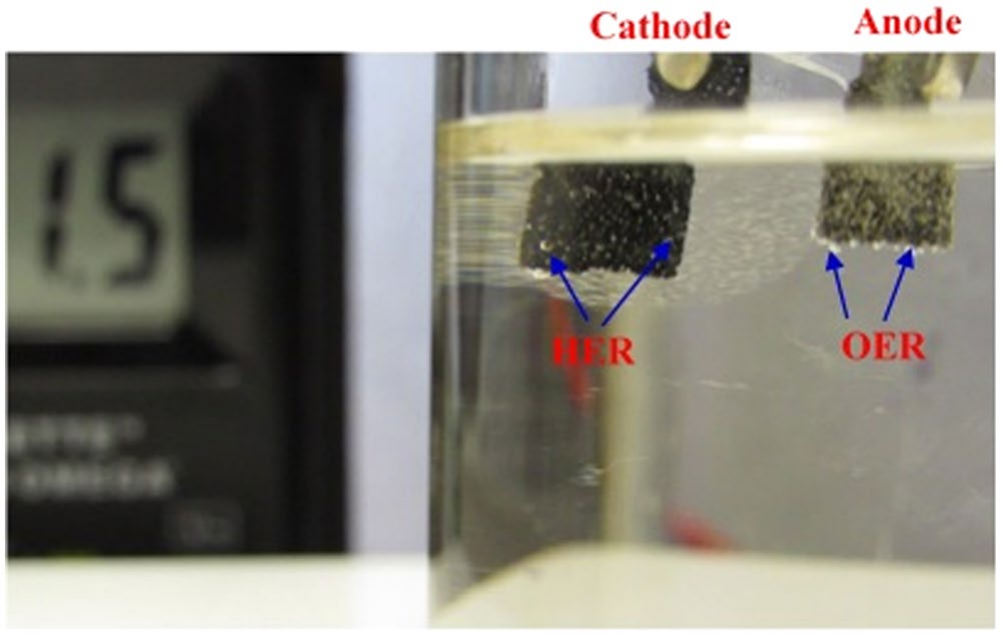
Demonstration of water-splitting device driven by a DC power supply at a cell voltage of 1.5 V to deliver a current density of ~6 mA cm^−2^.

Since NiSe_2_ shows high catalytic activity for both OER and HER processes in alkaline medium, we have designed a full water splitting system using NiSe_2_ for both anode and cathode. This bifunctional catalyst exhibits high performance towards overall water splitting and could deliver a current density of ~6 mA cm^−2^ with an applied cell voltage of 1.5 V in 1 M KOH (Fig. 9) during which bubbles of O_2_ and H_2_ were steadily released at both electrodes. It should be noted here that although the thermodynamic water splitting voltage is 1.23 V, it is reckoned that an ideal electrolyzer should be able to split water at 1.48 V for maximum efficiency without any heat exchange with the surrounding, also referred to as the thermoneutral potential^54, 55^. In the present case, water electrolysis was carried out in a closed electrochemical cell containing NiSe_2_ as the anodic and cathodic catalyst without any external source of heat. Electrolysis energy efficiency is calculated as the ratio between 1.48 V and the water splitting cell voltage. Electrolysis energy efficiency of the NiSe_2_ catalyst was calculated to be ~100% (Supplementary Table 2) at the onset potential of electrolysis (1.43 V), while at higher current densities (100 mA cm^−2^) the efficiency was 83%. This was compared with the energy efficiency obtained from an electrolyzer containing Pt as the cathode catalyst for HER and RuO_2_ as the anodic catalyst for OER, which gave a cell voltage of 1.83 V at 100 mA cm^−2^ (81% efficiency). In comparison with the reported literature, as a bifunctional catalyst, NiSe_2_ exhibits the highest electrolysis energy efficiency obtained so far under ambient conditions and is closest to the elusive most efficient and green water electrolyzer.

## Conclusions

In summary, we have electrodeposited nanostructured NiSe_2_ films as an efficient bifunctional electrocatalyst for overall water splitting with energy efficiency exceeding 83% at high current density. As-deposited NiSe_2_ reported here shows the lowest onset potential and overpotential at 10 mA cm^−2^ amongst all the Ni-based OER electrocatalysts (Supplementary Table 3). Through electrochemical measurements and structural characterization, we have proven that this enhancement in primarily due to lowering of the oxidation potential of Ni^2+^ to Ni^3+^ that in turn is a consequence of changing the oxide lattice to the more covalent selenide lattice, as well as the preferential growth direction of the film which exposes a Ni-rich surface as the terminating lattice plane.

## Electronic supplementary material

Supporting Information
