# Supplementary material for: Textured NiSe2 Film: Bifunctional Electrocatalyst for Full Water Splitting at Remarkably Low Overpotential with High Energy Efficiency
Source: Sci Rep. 2017 May 25;7:2401. doi: 10.1038/s41598-017-02285-z (PMC5445097; doi:10.1038/s41598-017-02285-z)
Supplement: Supplementary file 1 — Supporting Information [file 41598_2017_2285_MOESM1_ESM.pdf]

Textured $NiSe_2$ Film: Bifunctional Electrocatalyst for Full Water Splitting at Remarkably Low Overpotential with High Energy Efficiency

*Abdurazag T. Swesi,[1] Jahangir Masud,[1] Wipula P. R. Liyanage,[1] Siddesh Umapathi,[1] Eric Bohannan,[2] Julia Medvedeva[3], Manashi Nath[1]**

[1]Department of Chemistry, Missouri University of Science and Technology, Rolla, MO 65409.

[2]Materials Research Center, Missouri University of Science and Technology, Rolla, MO 65409.

[3]Department of Physics, Missouri University of Science and Technology, Rolla, MO 65409.

*E-mail: nathm@mst.edu

Experimental & Methods

Materials were reagent grade and were used as received without further purification. Nickel sulfate hexahydrate $NiSO_4.6H_2O$ was purchased from Fisher Scientific Company and Sodium selenite (Na_2SeO_3), was purchased from Alfa Aeasar. Au-coated glass slide used as substrates in electrodeposition was purchased from Deposition Research Lab Incorporated (DRLI), Lebanon, Missouri.

Electrodeposition of $NiSe_2$

The electrocatalytic $NiSe_2$ films were prepared by direct electrodeposition on electrodes such as, Au-coated glass, glassy carbon (GC), carbon fiber paper (CFP), and Ni foam. The electrolytes were prepared using analytical grade reagents and deionized (DI) water with a resistivity of 18 MΩ·cm. Prior to electrodeposition, the substrates were cleaned by ultrasonic treatment in micro-90 detergent followed by rinse with isopropanol for three times and eventually rinsed with deionized water (15 min each step) to ensure clean surface. Au-coated glass was covered with a Teflon tape, leaving an exposed geometric area of 0.283 cm^2, and connected as the working electrode. An IviumStat potentiostat was used to control the electrodeposition process and to monitor the current and voltage profiles. $NiSe_2$ was electrodeposited on the substrate by a controlled-potential deposition at -1.2 V (*vs* Ag/AgCl) for 30 min at 40°C from an aqueous solution containing 63 mM $NiSO_4.6H_2O$ and 150 mM Na_2SeO_3 . The pH of the electrolytic bath was adjusted to ~ 2.5 with dilute HCl. After each electrodeposition, the electrodeposited films were washed with deionized water in order to remove impurities and adsorbents from the surface.

Synthesis of $NiSe_2$ by hydrothermal method[S1]:

$NiSe_2$ was synthesized by hydrothermal method. In a typical procedure, 5 mmols of $NiCl_2.6H_2O$ and 10 mmols of SeO_2 were mixed in 10 ml of deionized water. The solution was stirred on a magnetic stirrer. About 5 mins later 0.1 ml (0.3mols) of hydrazine monohydrate was added. The solution of stirred for another 5 mins and then transferred to 23 ml Teflon - lined autoclave, which was sealed and maintained at 145°C for 24 h and then naturally cooled to room temperature. The resulting black solid was then washed several times with DI water and ethanol. The solid was dried in an oven maintained at 40°C overnight.

Preparation of electrode:

4 mg of the powder NiSe2 was dispersed in 200 µL of 1% Nafion in ethanol. The mixture was sonicated for 30 mins to produce a homogeneous ink. 20 µL of the catalyst ink was drop casted on to the Carbon Fiber Paper (CFP) at room temperature. Finally the as prepared catalyst ink was allowed to dry at room temperature for 8 hrs.

Electrodeposition of $Ni(OH)_2$ [S2]

The nickel hydroxide was deposited on the Au/glass substrate from 0.08 M $Ni(NO_3)_2$ aqueous solution at a potential of -0.86 V vs. Ag|AgCl|$KCl_{(sat)}$ for 5 min.

Methods of Characterization

Powder X-ray Diffraction (XRD):

<u>Thin film (asymmetric) diffraction:</u>

Diffraction patterns were obtained with a Cu source utilizing a PANalytical X'Pert Materials Research Diffractometer, with a fixed incident angle of 1 degree. The incident beam optic module was an x-ray mirror (PW 3088/60, PANalytical), while the diffracted beam optic module was a 0.18 degree parallel plate collimator (PW 3098/18, PANalytical).

Symmetric diffraction (Figure 6):

Diffraction patterns were obtained with a Cu source utilizing a PANalytical X'Pert Materials Research Diffractometer in gonio mode with incident and diffracted angles remaining equal to one another as the diffraction pattern was obtained. The incident beam optic module was a hybrid monochromator (PW 3147/00, PANalytical), consisting of an x-ray mirror and a 2-crystal Ge(220) 2-bounce monochromator. The diffracted beam optic module was a 0.18 degree parallel plate collimator (PW 3098/18, PANalytical).

The average catalyst particle size was calculated from the XRD diffraction peak width using the Scherrer equation:[S3]

$$L = \frac{K\lambda}{\beta \mathrm{Cos}\theta} \qquad (1)$$

where L is the particle size, λ is the X-ray wavelength in nanometer (0.15418 nm), β is the peak width of the diffraction peak profile at half maximum height in radians and *K* is a constant, normally taken as 0.9.

Scanning Electron Microscopy (SEM): SEM image of the modified electrode surfaces was obtained using a FEI Helios NanoLab 600 FIB/FESEM at an acceleration voltage of 10 kV and a working distance of 4.8 mm. Energy dispersive spectroscopy (EDS) along with line scan analysis was also obtained from the SEM microscope.

X-ray Photoelectron Spectroscopy (XPS): XPS measurements of the catalysts were performed by KRATOS AXIS 165 X-ray Photoelectron Spectrometer using monochromatic Al X-ray source. The spectra were collected as is and after sputtering with Ar for 2 min which removes approximately 2 nm from the surface.

Raman Spectra: Horiba Jobin Yvon Lab Raman ARAMIS model was used to perform Raman microspectroscopy on the as-deposited catalyst films. The laser used was He-Ne with a power of about 1.7 mW over a range of 100 – 2000 cm^{-1}. The spectra were iterated over an average of 25 scans.

Electrochemical Characterization and Catalytic Studies:

The OER catalytic performance was estimated from linear scan voltammetry (LSV) plots while the stability of the catalyst was studied by chronoamperometry. All electrochemical measurements in this work were performed in a three-electrode system with an IviumStat potentiostat using Ag|AgCl|$KCl_{(sat.)}$ and Pt mesh as reference and counter electrodes, respectively unless otherwise noted. The Ag|AgCl|$KCl_{(sat.)}$ reference electrode was calibrated by measuring open circuit potential (OCP at -0.199V) at Pt wire in pure H_2 saturated 1.0 M H_2SO_4 solution. The measured potentials *vs* the Ag/AgCl at any given pH is were converted to the reversible hydrogen electrode (RHE) scale *via* Nernst equation (eq. 2):

$$E_{\mathrm{RHE}} = E_{\mathrm{Ag|AgCl}} + 0.059\mathrm{pH} + E^{\mathrm{o}}_{\mathrm{Ag|AgCl}} \qquad (2)$$

where E_{RHE} is the converted potential vs. RHE, $E_{\mathrm{Ag|AgCl}}$ is the experimentally measured potential against Ag|AgCl reference electrode, and $E^{o}_{\mathrm{Ag|AgCl}}$ is the standard potential of Ag|AgCl at 25 ^{o}C (0.199 V). For most of the electrochemical characterizations, the electrode area of the film surface was kept constant at 0.283 cm^2.

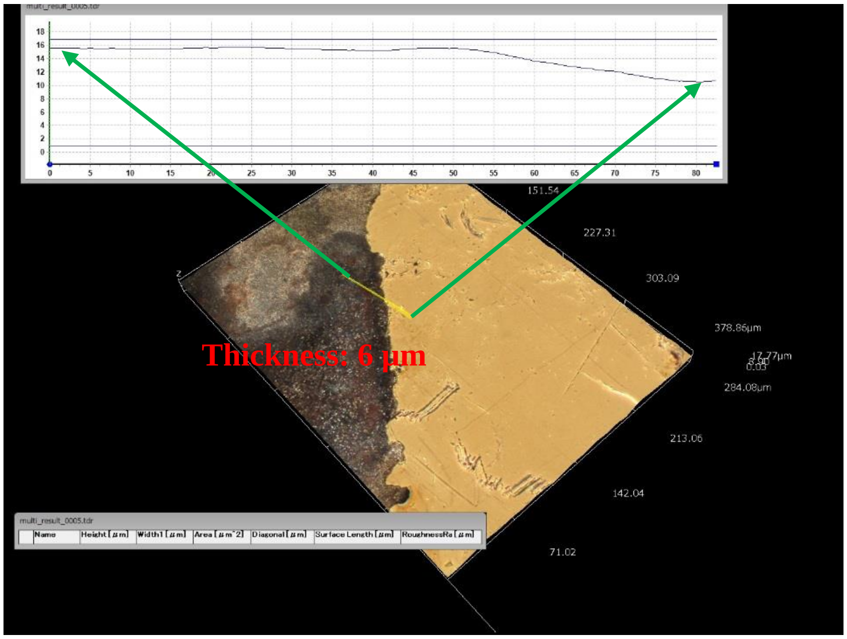

Supplementary Figure 1. 3D imaging and 3D profile of as prepared $NiSe_2$. The HIROX KH-8700 Digital Microscope, is the next generation system for the high precision of measurement and 3D profiling, was used to measure the approximate thickness of electrodeposited $NiSe_2$

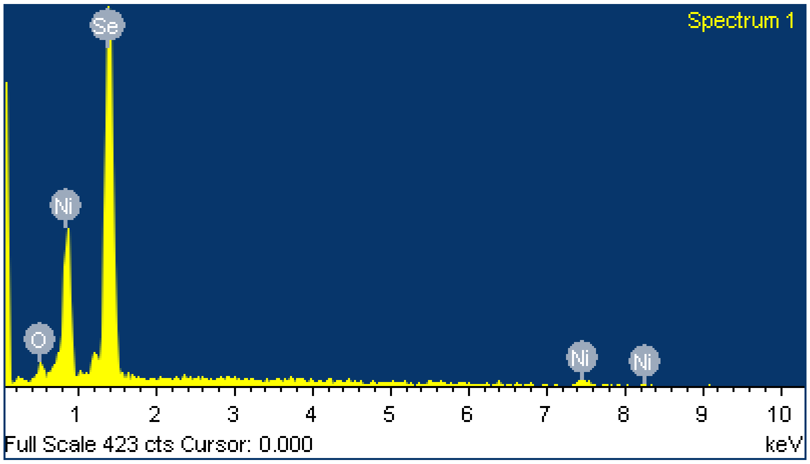

Supplementary Figure 2. EDS spectrum of as prepared $NiSe_2$.

Atomic ratio of Nickel and selenium

Element	Atomic%	Ni : Se
O K	4.25	
Ni L	31.30	1.0 : 2.05
Se L	64.45	
Totals	100	

Electrochemical active area (ECSA) of catalyst

The ECSA of the $NiSe_2$ can be estimated by measuring the electrochemical capacitance of the electrode–electrolyte interface in the double-layer regime of the voltammograms. To estimate ECSA of the catalysts in a N_2 saturated 1 M KOH solution, the electrode was potentiostatically cycled from -0.34 to -0.27 V *vs*. Ag|AgCl at scan rates between 2.5 and 40 mV s^{-1} (Supplementary Figure S3). The capacitive current (i_{DL}) was evaluated from the non-faradaic double-layer region. This current indeed is proportional to the scan rate, v (V s^{-1}) as shown in eqn (3):

$$i_{DL} = C_{DL} \times v \qquad (3)$$

where C_{DL} is the specific capacitance of the electrode double layer (F cm^{-2} electrode).

Plotting of i_{DL} against v gives the value of C_{DL}. The ECSA of the catalyst was estimated from the double-layer capacitance from the following eqn (4):

$$\mathrm{ECSA} = C_{DL}/C\mathrm{s} \qquad (4)$$

*C*s is the specific capacitance which is reported from 0.022 to 0.130 mF cm^{-2} in alkaline solution.[S3] Here, we use the value of *C*s is 0.040 mF cm^{-2} based on previously reported Ni-based OER catalysts.[S3] The ECSA and the roughness factor (RF) of the $NiSe_2$ was calculated as 4.7 cm^2 and 16.6, respectively.

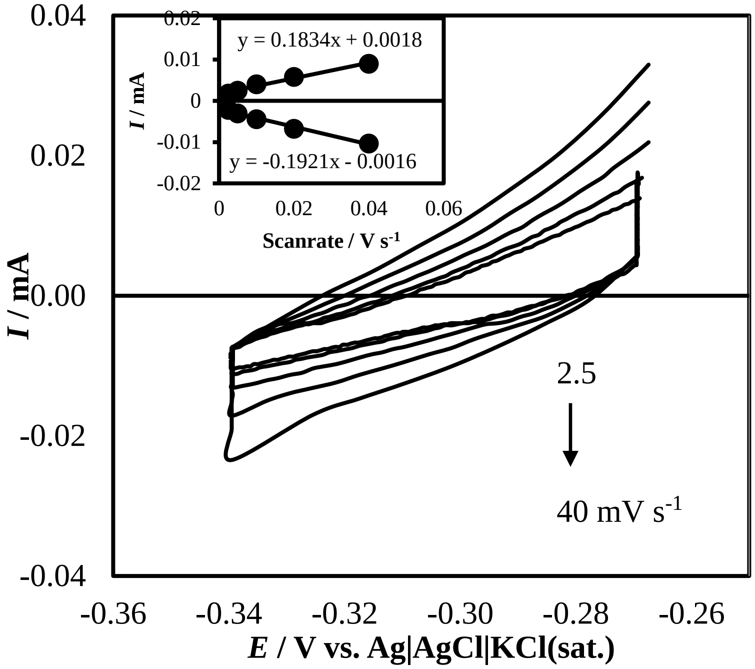

Supplementary Figure 3. Cyclic voltammograms measured for $NiSe_2$ catalyst in N_2 saturated 1.0 M KOH solution at different scan rate from 2.5 to 40 mV s^{-1}. Inset shows plot of anodic and cathodic current measured at -0.30 V as function of scan rate.

Estimation of Onset potential and overpotential to achieve 10 mA cm^{-2}

In case of LSV at $NiSe_2$ catalyst, the onset of OER activity and overpotential at 10 mA cm^{-2} was heavily masked by the large pre-oxidation peak. Hence, to get a better idea about the onset potential for oxygen evolution, we have carried out cyclic voltammograms (CVs) between 1.0 to 1.45 V *vs* RHE. The cyclic voltammogram for an as-deposited film in 1 M KOH (Supplementary Figure S4) exhibits a redox couple; $Ni^{2+} \rightarrow Ni^{3+}$ at 1.34 V *vs* RHE and $Ni^{3+} \rightarrow Ni^{2+}$ at 1.22 V *vs* RHE. [S4] A more accurate onset potential for water oxidation and overpotential at 10 mA cm^{-2} was obtained from the reverse plot in the CV. Accordingly it was observed that a current density of 10 mA cm^{-2} was obtained at an overpotential of 140 mV, which is the lowest value reported till date amongst all the known OER electrocatalysts.

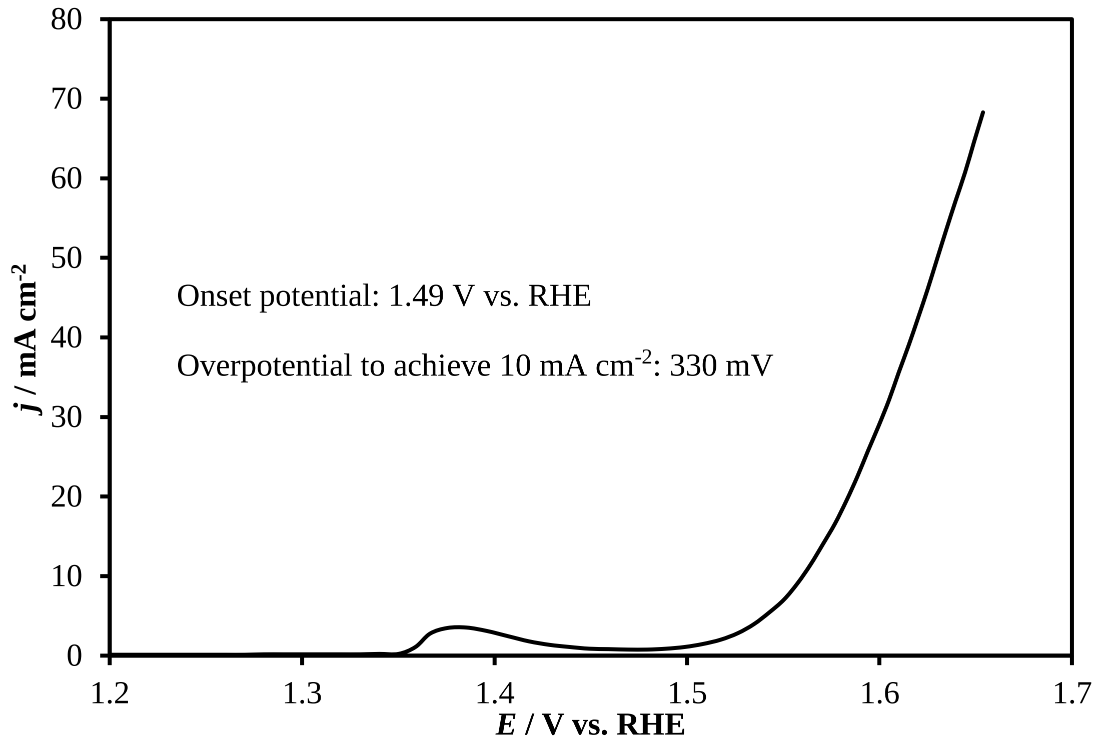

Supplementary Figure 4: LSV for OER at $Ni(OH)_2$ / Au-glass in N_2-saturated 1.0 M KOH at a scan rate of 10 mV s^{-1}.

Testing of evolved gas and Faradic efficiency

An experiment was designed to probe that the bubbles formed at the anode were O_2. A series of ORR experiments were conducted after various periods of O_2 generation. The electrolyte was blanketed with a N_2 atmosphere to get rid of dissolved O_2 during measuring the ring current. The disk electrode was held at constant potential of 1.37 V vs RHE to deliver 10 mA cm^{-2} to ensure O_2 generation for various times 0.5, 1 and 5 h in 1 M KOH solution. After each OER run, the evolved oxygen was reduced at Pt electrode as shown in Figure S7a. It was observed that the ORR current increased as the generation time of O_2 increased. The highest current was after 5 h O_2 generation which was comparable to that obtained from a solution saturated by passing gaseous O_2. After 5 h generation of O_2, the same solution was purged again with N_2 for 2 h to remove all the dissolved O_2. The ORR current measured after N_2 saturation showed minimal current as was observed before start of the experiment. This proves that the source of O_2 was indeed from the anode where O_2 was evolved due to the electrocatalytic process.

The RRDE experiment was employed to determine the OER Faradaic efficiency for the $NiSe_2$ electrocatalyst. $NiSe_2$ was deposited on glassy carbon (GC) which was connected as the disk electrode in a RRDE set-up with Pt as the ring electrode. The applied disk potential was held at several voltages in the OER kinetic-limited region for 1 min, while being rotated at a 1600 rpm under a pure N_2 gas blanket. The ring current was collected at 0.2 V applied ring potential (*vs.* RHE) to reduce the produced oxygen from the disk (anode) sufficiently. The Faradaic efficiency was calculated as below:

$$\text{Faradaic Efficiency} = \frac{2i_r}{i_d\,N} \qquad (6)$$

where i_r and i_d are the measured ring and disk currents, respectively, and N is the collection efficiency of RRDE, which was measured as ~ 0.24 in this work. Figure S7b shows the plot of the disk current as a function of the ring current and Faradaic efficiency over the $NiSe_2$ catalyst in 1.0 M KOH. The maximum Faradaic efficiency was observed at about 99.5 % at the applied disk potential of 1.4 V (*vs*. RHE), which is corresponding to about 1.0 mA cm^{-2} disk current density. As the disk voltage increased to 1.46 V (*vs*. RHE), the Faradaic efficiency decayed to 50.2 %. This decrease could be attributed to large amounts of oxygen being produced at the disc electrode which cannot be completely reduced efficiently at the Pt ring electrode.[S5]

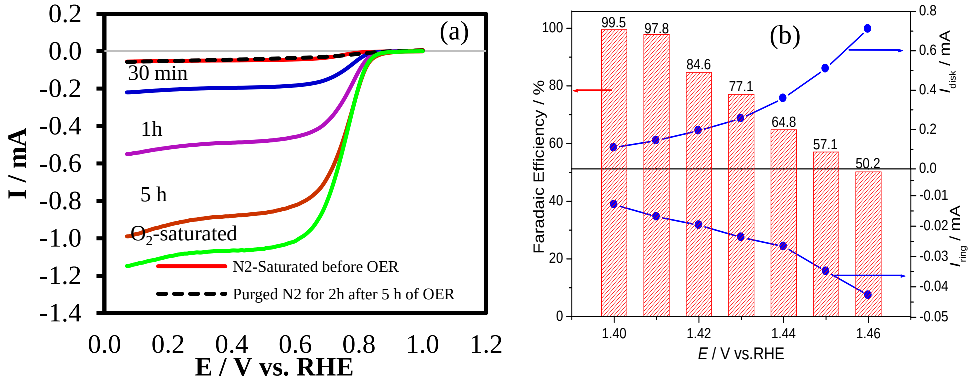

Supplementary Figure 5. (a) Electrochemical evidence of O_2 generation at the disk electrode and reduction at ring electrode when constant current of 10 mA cm^{-2} was applied at disk electrode for varying periods of time (0.5, 1 and 5 h) in N_2 saturated and blanketed 1.0 M KOH. (b) Faradaic efficiency of catalyst in 1.0 M KOH at 1600 rpm under N_2 saturation. The disk and ring currents of RRDE plotted as function of the applied disk potential along with the calculated Faradaic efficiency.

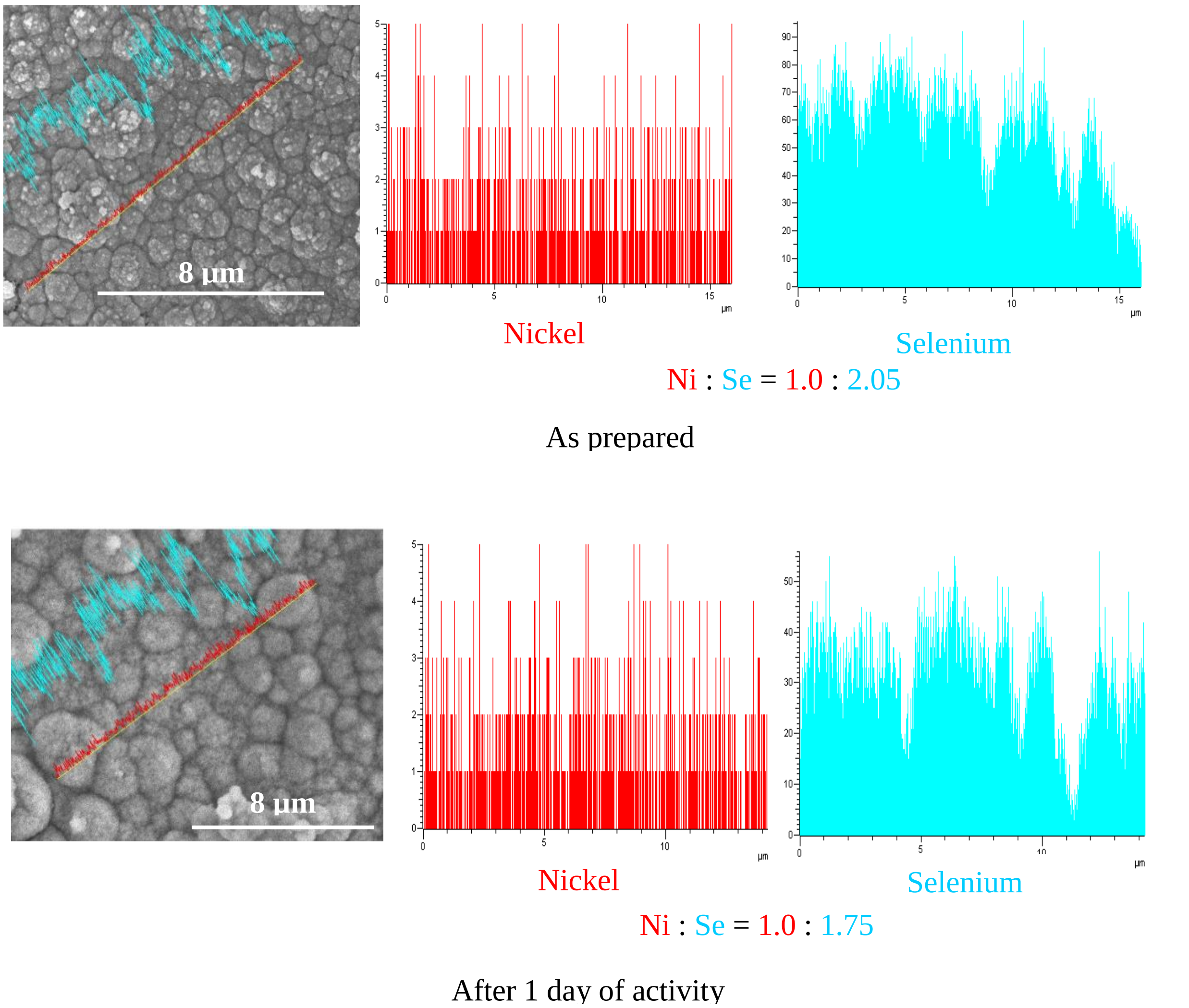

Supplementary Figure 6. SEM images and EDS line scanning of $NiSe_2$ before and after OER catalytic activity.

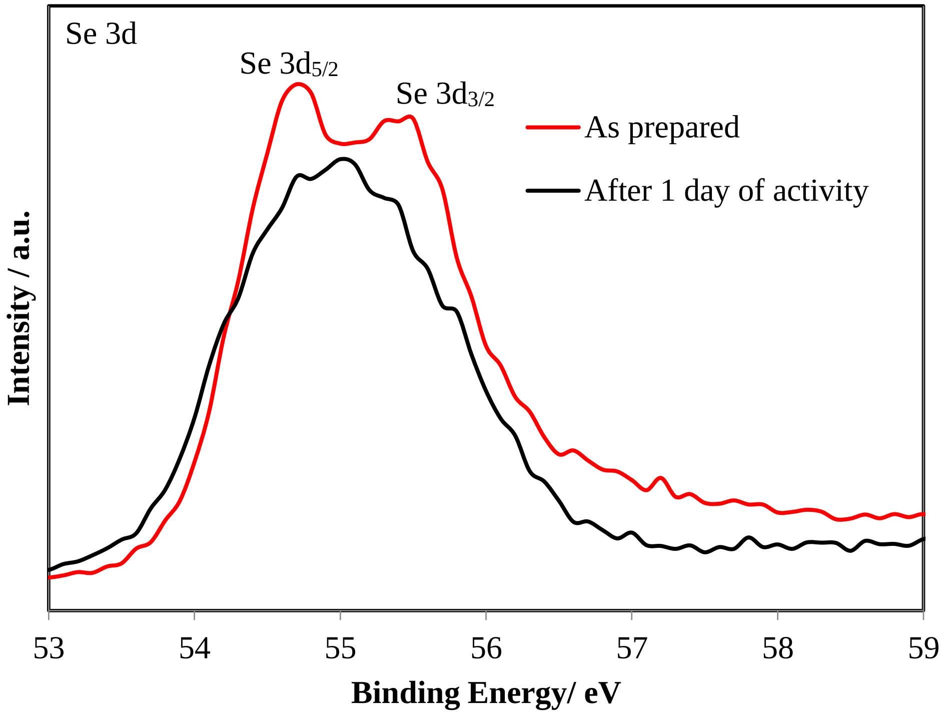

Supplementary Figure 7. XPS spectra of Se 3d peaks obtained from the catalyst before and after chronoamperometry (24h).

O1s XPS spectra of $NiSe_2$:

The O 1s signals in as prepared $NiSe_2$ (Figure S8a) is largely due to surface-adsorbed oxygen which may be chemisorbed as due to exposure to atmosphere.[S6] There is no evidence for formation

of Ni-oxide on the surface (no peak at 528.5-529.0 eV)[S7] of the as prepared catalyst. The deconvoluted O1s XPS spectrum after 24 h of OER catalyst after activity, as shown in Figure S8b, revealed that it could be fitted into three contributions attributed to chemisorbed oxygen (530.8 eV) [S6], surface-absorbed SeO_x (532.9 eV),[S8] and physisorbed and chemisorbed water at or near the surface (535.0 eV).[S8] The presence of Se-oxides is very common in most metal selenides. Interestingly, this spectrum also reveals the absence of XPS peaks corresponding to Ni-O even after prolonged catalytic activity.

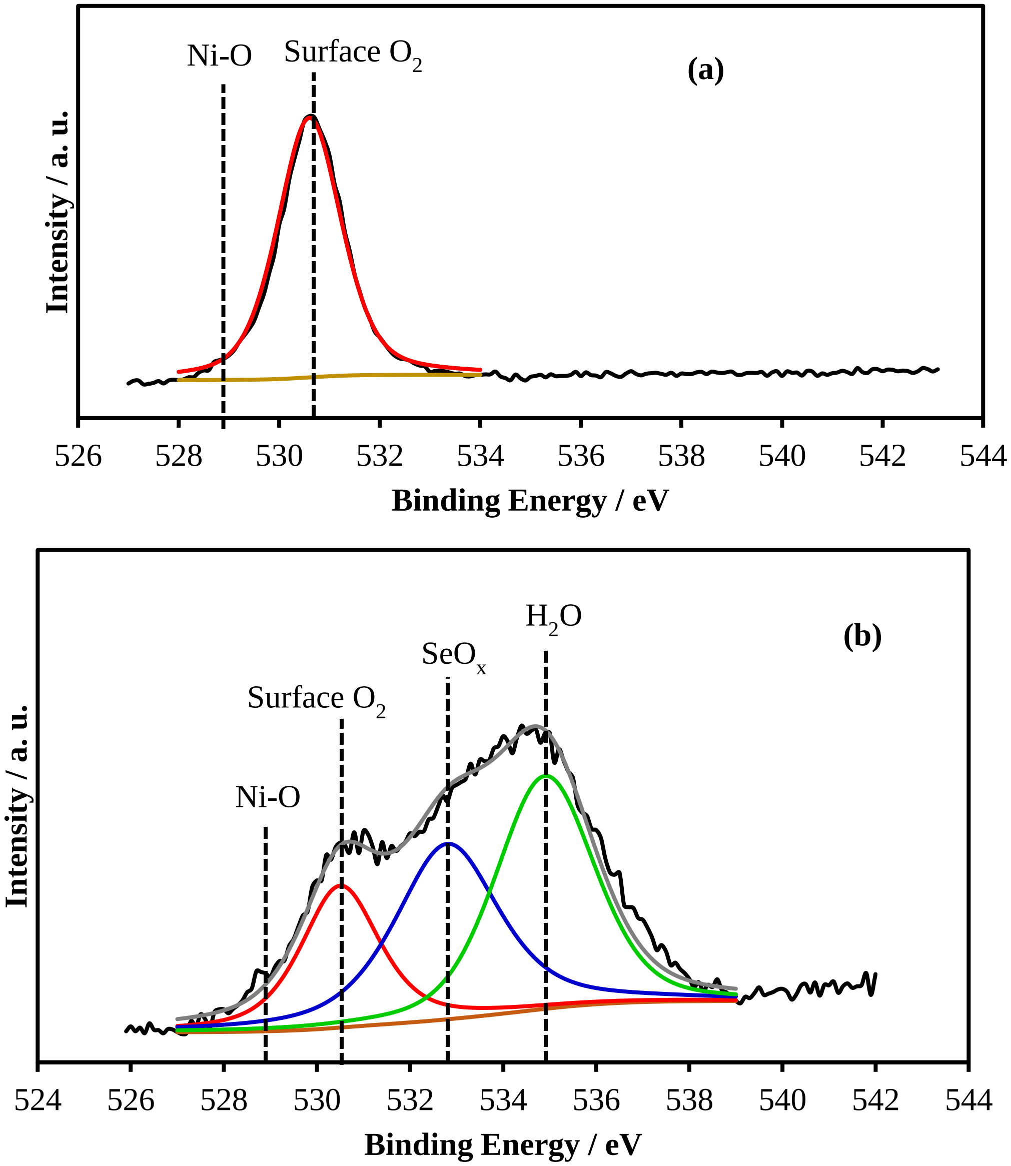

Supplementary Figure 8. XPS spectra of O 1s peaks obtained from the catalyst (a) before and (b) after chronoamperometry.

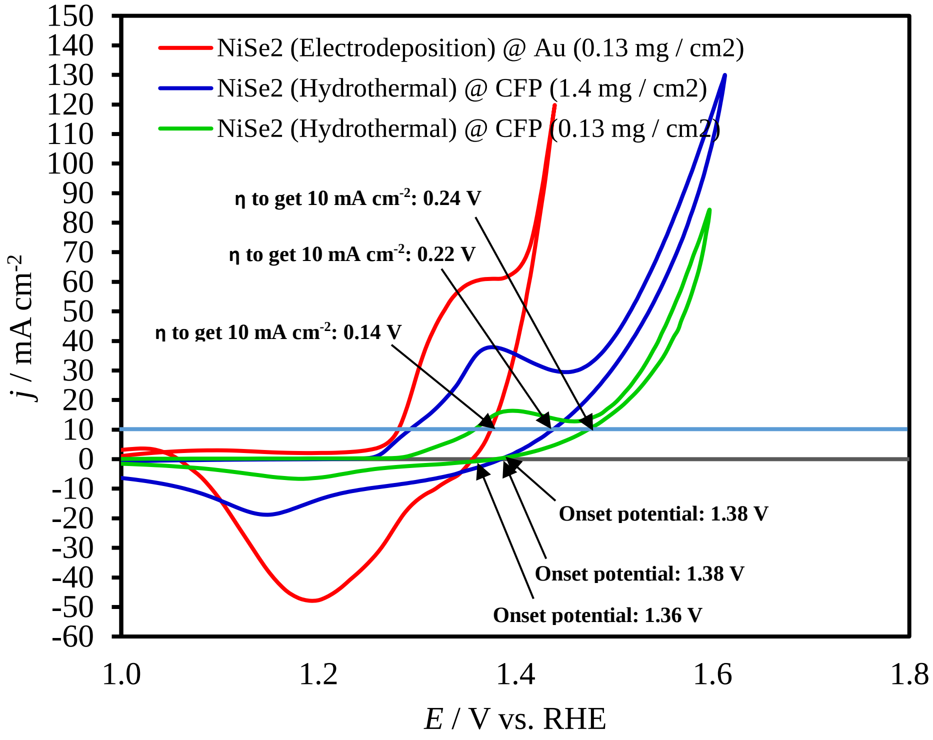

Supplementary Figure 9: Cyclic voltammogram for OER at $NiSe_2$ @ Au and $NiSe_2$ (Powder) @ CFP with variable loading of catalyst in N_2-saturated 1.0 M KOH at a scan rate of 10 mV s^{-1}.

H_2 gas evolution testing:

To confirm the H_2 evolution (HER) at $NiSe_2$ catalyst, a series of hydrogen oxidation reaction (HOR) experiments were performed after various periods of H_2 generation. First, HOR was carried out at Pt electrode in N_2 saturated and blanketed 1M KOH solution as a blank (Figure S11). Then, HER were conducted at constant potential of -0.2 V vs RHE for various times 0.5, 1 and 3 h in 1 M KOH solution. After each HER run, the evolved hydrogen was oxidized at Pt electrode (Figure S11). It was observed that the HOR current increased as the generation time of H_2 increased. The electrolyte solution was purge and saturated H_2 gas and again HOR experiment was performed at Pt electrode which was confirmed the reliability of experiment. Finally the same solution was purged again with N_2 for 2 h to remove all the dissolved H_2 and HOR was carried out that showed minimal current as was observed before start of the experiment. This series of experiments confirm H_2 evolution at $NiSe_2$ / Au electrode.

Catalytic Hydrogenation of *para*-nitrophenol (PNP) to *para*-aminophenol (PAP)

H_2 gas was further confirm by well-established catalytic reduction of para-nitrophenol (PNP) to para-aminophenol (PAP) and conversion was monitored spectrophotometrically. In this process, 100 µM PNP was dissolved in the 1 M KOH electrolyte and the working electrode (Au-glass coated with $NiSe_2$) was maintained at a constant potential of -0.3 V *vs* RHE, with Pt as counter and Ag|AgCl as reference electrode. Copious quantities of gas bubbles were observed to be evolved from the surface of the working electrode. Aliquots were collected at every 15 min and the UV-Vis spectra of the aliquot was measured. The absorbance of pure PNP and PAP were collected separately in 1 M KOH solution (Fig. 10). Two separate control experiments were performed in which the bare Au-glass electrode was maintained at -0.3 V (*vs* RHE) for up to 5h. In the second control experiment, the working electrode ($NiSe_2$@Au-glass) was maintained at

zero applied potential and H_2 gas was bubbled through the solution for 5 h. In both of these cases the absorbance of PNP did not show any appreciable change even after 5 h.

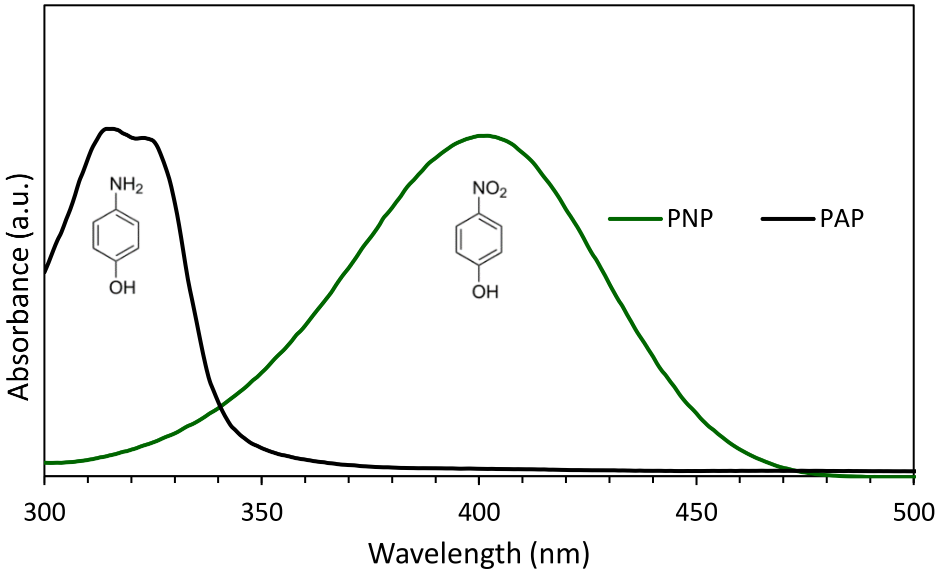

Supplementary Figure 10. UV-Vis spectra of *para*-nitrophenol (PNP) and *para*-aminophenol (PAP).

HER Faradaic Efficiency:

The Faradic efficiency of a HER catalyst is defined as the ratio of the amount of H_2 evolved during the catalytic experiments to the amount of H_2 expected based on theoretical considerations. To measure the Faradic efficiency of HER, we carried out the following experiment. We collected the evolved H_2 gas (at constant potential of -0.5 V vs. RHE for 1h) by water displacement method,[S9] and then calculated the moles of H_2 generated from the reaction with an ideal gas law. As for the theoretical value, we assumed that the current produced was solely due to HER process at the

working electrode, based on the coupled HOR reaction. The theoretical amount of H2 was then calculated by applying Faraday law.

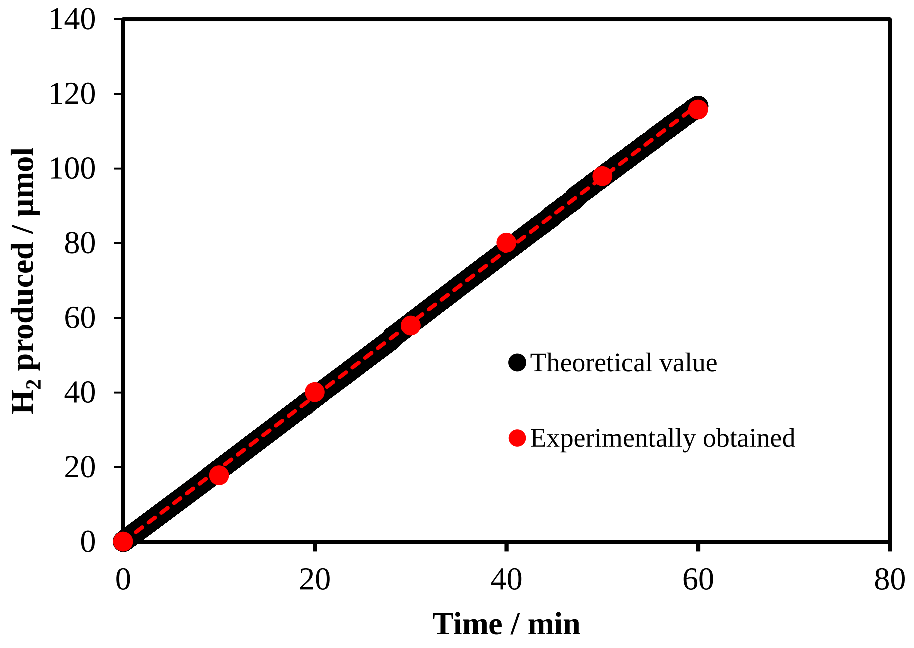

Supplementary Figure 11: HER faradaic efficiency of $NiSe_2$ catalyst where H_2 was evolved at a potential of -0.5 V vs. RHE for 1h and compare the theoretically calculated and experimentally obtained H_2 gas produced.

Supplementary Table 1: Comparison of OER activity at different nickel selenide based catalysts with loading.

Catalyst	Loading	Activity	References
$NiSe_2$	1 mg / cm^{2}	250 mV (10 mA cm^{-2})	*ACS Appl. Mater. Interfaces* 2016, **8**, 5327.
Co–Ni–Se/C/NF	1.5 mg / cm^{2}	275 mV (30 mA cm^{-2})	*J. Mater. Chem. A* 2016, **4**, 15148
Fe–NiSe/FeNi foam	4.8 mg / cm^{-2}	245 mV (50 mA cm^{-2})	*Chem. Commun*. 2016, **52** ,4529
Co-doped $NiSe_2$	1.67 mg / cm^{-2}	320 mV (100 mA cm^{-2})	*Nanoscale* 2016, **8**, 3911
$(Ni, Co)_{0.85}Se$	5 mg / cm^{2}	300 mV (97.5 mA cm^{-2})	*Adv.Mater.* 2016, DOI: 10.1002/adma.201503906
NixFe1-xSe2-DO	4.1 mg / cm^{-2}	195 mV (10 mA cm^{-2})	*Nat. Commun*. 2016, **7**, 12324
$NiSe_2$-DO	4.1 mg / cm^{-2}	241 mV (10 mA cm^{-2})	
Ni_3Se_2 –Cu foam	3 mg / cm^{2}	284 mV (10 mA cm^{-2})	*Catal. Sci. Technol* 2015, **5**, 4954.
NiSe- Ni foam	2.8 mg / cm^{2}	251 mV (10 mA cm^{-2})	*Angew. Chem.Int. Ed.* 2015, **54**, 9351
$NiSe_2$ (Electrodeposition)	0.13 mg / cm^{2}	140 mV (10 mA cm^{-2})	This manuscript
$NiSe_2$ (Hydrothermally)	1.4 mg / cm^{2}	220 mV (10 mA cm^{-2})	
$NiSe_2$ (Hydrothermally)	0.13 mg / cm^{2}	240 mV (10 mA cm^{-2})	

Supplementary Table 2 Calculation of energy efficiency of water electrolyzer (%)

	OER (V)[a]		HER (V)[a]		Water splitting cell voltage		Energy efficiency (%)[b]	
	at 10 $mA.cm^{-2}$	at 100 $mA.cm^{-2}$	at 10$mA.cm^{-2}$	at 100 $mA.cm^{-2}$	at 10 $mA.cm^{-2}$	at 100 $mA.cm^{-2}$	10 $mA.cm^{-2}$	100 $mA.cm^{-2}$
Pt	-	-	0.06	0.12	1.61	1.83	92	80.9
RuO_2	1.55	1.71	-	-				
$NiSe_2$	1.37	1.43	0.17	0.35	1.54	1.78	96.1	83.1

[a]All voltages are *vs* RHE.

[b]Energy efficiency of water electrolysis≅ (1.48 V/E_{cell}) ×100.

Supplementary Table 3 Comparison of OER activity at different catalysts.

Electrocatalyst	Electrolyte	Overpotential (mV *vs* RHE) @ 10[a], 20[b] or 50[c] mA cm^{-2}	Reference
**$NiSe_2$/Au**	**1 M KOH**	**140[a], 150[b] & 170[c]**	**This work**
**$NiSe_2$ (powder) / CFP**		**220[a], 260[b] & 330[c]**	
Ni_3Se_2 / Au	0.3 M KOH	290[a]	S5
NiSe/NiF	1 M KOH	270[a]	S10
Fe-doped NiSe	1 M KOH	264 mV (100 mA cm^{-2})	S11
$Co_{0.85}Se$	1 M KOH	324[a]	S12
$(Ni,CO)_{0.85}Se$	1 M KOH	255[a]	
$Co_{0.13}Ni_{0.87}Se_2$/Ti	1 M KOH	320 mV (100 mA cm^{-2})	S13
$NiSe_2$ /Ti	1 M KOH	350 mV (100 mA cm^{-2})	
$NiSe_2$	1 M KOH	250[a]	S14
$CoSe_2$	1 M KOH	430[a]	
NiS/ Ni foam	1 M KOH	335[c]	S15
Ni_3Se_2/Cu foam	1 M KOH	343[c]	S16
$Ni_xFe_{1-x}Se_2$-DO	1M KOH	195[a]	S17
$NiSe_2$-DO	1M KOH	241[a]	
CoSe/Ti mesh	1 M KOH	341[c]	S18
NiFe LDH/NF	1 M KOH	269[b] & 349[c]	S19
$Ni(OH)_2$	1 M KOH	313[b]	S20
NiO nanoparticles	1 M KOH	347[b]	
NiO_x/C	1 M KOH	335[a]	S21
($Ni_{0.69}Fe_{0.31}O_x$/C)	1 M KOH	280[a]	
NiOOH	1 M KOH	525[a]	S22
Amorphous NiO	1 M KOH	>470 [b]	S23
$NiCo_2O_4$	1 M KOH	391[b]	S24
Ni/Ni_3N foam	1 M KOH	399[b]	S25
NiCo LDH	1 M KOH	393[b]	S26
Ni-Co-O@Ni-Co-S NA	1 M KOH	300[b]	S27
CQDs/NiFe-LDH	1 M KOH	271[b]	S28
Ni_5P_4/Ni foil	1 M KOH	363[c]	S29
Ni–P/Cu foam	1 M KOH	410[c]	S30
NiMo HNRs/Ti mesh	1 M KOH	344[c]	S31

References

S1. Sobhani, A. & Niasari, M. S. Synthesis and characterization of a nickel selenide series via a hydrothermal process *Superlattices and Microstructures* **65**, 79-90 (2014).

S2. Wu, H. Y. & Wang, H. W. Electrochemical synthesis of nickel oxide nanoparticulate films on nickel foils for high-performance electrode materials of supercapacitors. *Int. J. Electrochem. Sci.* **7**, 4405 – 4417 (2012).

S3. Masud, J.; Nguyen, T. V.; Singh, N.; McFarland, E.; Ikenberry, M.; Hohn, K.; Pan, C. J.; Hwang, B. J. A RhxSy/C catalyst for the hydrogen oxidation and hydrogen evolution reactions in HBr. *J. Electrochem. Soc.* **162**, F455-F462 (2015).

S4. Louie, M. W. & Bell, A. T. An investigation of thin-film Ni-Fe oxide catalysts for the electrochemical evolution of oxygen *J. Am. Chem. Soc*. **135**, 12329 – 12337 (2013).

S5. Swesi, A. T.; Masud, J. & Nath, M. Nickel selenide as a high-efficiency catalyst for oxygen evolution reaction. *Energy Environ. Sci*. **9**, 1771-1782 (2016).

S6. Parkinson, C. R.; Walker, M. & McConville, C. F. Reaction of atomic oxygen with a Pt(111) surface: chemical and structural determination using XPS, CAICISS and LEED. *Surf. Sci.* **545**, 19-33 (2003).

S7. Khawaja, E.E.; Salim, M.A.; Khan, M. A.; Al-Adel, F.F.; Khattak, G.D. & Hussain, Z. XPS, auger, electrical and optical studies of vanadium phosphate glasses doped with nickel oxide. *J. Non-cryst. Solids* **110**, 33 (1989).

S8. Xia, C.; Jiang, Q.; Zhao, C.; Hedhili, M. N. & Alshareef, H. N. Selenide-Based Electrocatalysts and Scaffolds for Water Oxidation Applications. *Adv. Mater*. **28**, 77–85 (2016).

S9. Wang, J.; Xia, H.; Peng, Z.; Lv, C.; Jin, L.; Zhao, Y.; Huang, Z. & Zhang, C. Graphene porous foam loaded with molybdenum carbide nanoparticulate electrocatalyst for effective hydrogen generation. *ChemSusChem* **9**, 855-862 (2016)

S10. Tang, C.; Cheng, N.; Pu, Z.; Xing, W. & Sun, X. NiSe nanowire film supported on nickel foam: an efficient and stable 3D bifunctional electrode for full water splitting. *Angew. Chem. Int. Ed*. **54**, 9351 –9355 (2015).

S11. Tang, C.; Asiri, A. M. & Sun, X. Highly-active oxygen evolution electrocatalyzed by a Fe-doped NiSe nanoflake array electrode. *Chem.Commun*. **52**, 4529-4532 (2016).

S12. Xia, C.; Jiang, Q.; Zhao, C.; Hedhili, M. N. & Alshareef, H. N. Selenide-based electrocatalysts and scaffolds for water oxidation applications. *Adv. Mater.* **28**, 77–85 (2016).

S13. Liu, T.; Asiri, A. M. & Sun, X. Electrodeposited Co-doped $NiSe_2$ nanoparticles film: a good electrocatalyst for efficient water splitting. Nanoscale **8**, 3911- 3915 (2016).

S14. Kwak, I. H.; Im, H. S.; Jang, D. M.; Kim, Y. W.; Park, K.; Lim, Y. R.; Cha, E. H. & Park, J. $CoSe_2$ and $NiSe_2$ nanocrystals as superior bifunctional catalysts for electrochemical and photoelectrochemical water splitting. *ACS Appl. Mater. Interfaces* **8**, 5327−5334 (2016).

S15. Zhu, W.; Yue, X.; Zhang, W.; Yu, S.; Zhang, Y.; Wang, J. & Wang J. Nickel sulfide microsphere film on Ni foam as an efficient bifunctional electrocatalyst for overall water splitting. *Chem. Commun*. **52**, 1486-1489 (2016).

S16. Shi, J.; Hu, J.; Luo, Y.; Sun, X. & Asiri, A. Ni_3Se_2 film as a non-precious metal bifunctional electrocatalyst for efficient water splitting. *Catal. Sci. Technol*. **5**, 4954-4958 (2015).

S17. Xu, X.; Song, F. & Hu, X. A nickel iron diselenide-derived efficient oxygen-evolution

catalyst. *Nat. Commun*. **7**, 12324 (2016)

S18. Liu, T.; Liu, Q., Asiri, A.; Luo, Y.; Sun, X. An amorphous CoSe film behaves as an active and stable full water-splitting electrocatalyst under strongly alkaline conditions. *Chem. Commun*. **51**, 16683-16686 (2015).

S19. Luo, J.; Im, J.-H.; Mayer, M.; Schreier, M.; Nazeeruddin, M.; Park, N.; Tilley, S.; Fan, H. & Gratzel, M. Water photolysis at 12.3% efficiency via perovskite photovoltaics and Earth-abundant catalysts. *Science* **345** 1593–1596 (2014).

S20. Stern, L. & Hu, X. Enhanced oxygen evolution activity by NiO_x and $Ni(OH)_2$ nanoparticles. *Faraday Discuss*. **176**, 363 –379 (2014).

S21. Qiu, Y.; Xin, L. & Li, W. Electrocatalytic oxygen evolution over supported small amorphous Ni–Fe nanoparticles in alkaline electrolyte. *Langmuir* **30**, 7893–7901 (2014).

S22. Klaus, S.; Cai, Y.; Louie, M.; Trotochaud, L. & Bell, A. T. Effects of Fe electrolyte impurities on Ni(OH)2/NiOOH structure and oxygen evolution activity. *J. Phys. Chem. C* **119**, 7243-7254 (2015).

S23. Kuai, L.; Geng, J.; Chen, C.; Kan, E.; Liu, Y.; Wang, Q. & Geng, B. A reliable aerosol-spray-assisted approach to produce and optimize amorphous metal oxide catalysts for electrochemical water splitting. *Angew. Chem. Int. Ed*. **53**, 7547–7551 (2014).

S24. Peng, Z.; Jia, D.; Al-Enizi, A.; Elzatahry, A. & Zheng, G. From water oxidation to reduction: homologous Ni–Co based nanowires as complementary water splitting electrocatalysts. *Adv. Energy Mater*. **5**, 1402031-1402038 (2015).

S25. Shalom, M.; Ressing, D.; Yang, X.; Clavel, G.; Fellinger, T. & Antonietti, M. Nickel nitride as an efficient electrocatalyst for water splitting. *J. Mater. Chem. A* **3**, 8171–8177 (2015).

S26. Liang, H.; Meng, F.; Acevedo, M.; Li, L.; Forticaux, A.; Xiu, L.; Wang Z. & Jin, S. Hydrothermal continuous flow synthesis and exfoliation of NiCo layered double hydroxide nanosheets for enhanced oxygen evolution catalysis. *Nano Lett*. **15**, 1421–1427 (2015).

S27. Xu, W.; Lu, Z.; Lei, X.; Li, Y. & Sun, X. A hierarchical Ni–Co–O@Ni–Co–S nanoarray as an advanced oxygen evolution reaction electrode. *Phys. Chem. Chem. Phys*. **16**, 20402–20405 (2014).

S28. Tang, D.; Liu, J.; Wu, X.; Liu, R.; Han, X.; Han, Y.; Huang, H.; Liu, Y. & Kang, Z. Carbon quantum dot/NiFe layered double-hydroxide composite as a highly efficient electrocatalyst for water oxidation. *ACS Appl. Mater. Interfaces* **6**, 7918–7925 (2014).

S29. Ledendecker, M; Calderon, S. K.; Papp, C.; Steinruck, H. P.; Antonietti, M. & Shalom, M. The synthesis of nanostructured Ni_5P_4 films and their use as a non-noble bifunctional electrocatalyst for full water splitting. *Angew. Chem. Int. Ed*. **127**, 12538–12542 (2015).

S30. Liu, Q.; Gu, S. & Li, C. Electrodeposition of nickel-phosphorus nanoparticles film as a Janus electrocatalyst for electro-splitting of water. *J. Power Sources* **299**, 342-346 (2015).

S31. Tian, J.; Cheng, N.; Liu, Q.; Sun, X.; He, Y.; Asiri A. Self-supported NiMo hollow nanorod array: an efficient 3D bifunctional catalytic electrode for overall water splitting. *J. Mater. Chem. A* **3**, 20056-20059 (2015).
